# Hundred‐Nanometer‐Thick Stretchable Liquid Metal Films for Ultra‐Conformal Bioelectrodes

**DOI:** 10.1002/advs.202512762

**Published:** 2025-10-23

**Authors:** Shiying Li, Shuai Yang, Jinyun Liu, Feng Xu, Yuanzhao Wu, Qi Zhang, Zidong He, Jie Shang, Changchen Shi, Yiwei Liu, Run‐Wei Li

**Affiliations:** ^1^ Ningbo Institute of Materials Technology and Engineering Chinese Academy of Sciences Ningbo 315201 P. R. China; ^2^ Center of Materials Science and Optoelectronics Engineering University of Chinese Academy of Sciences Beijing 100049 P. R. China; ^3^ Eastern Institute of Technology Ningbo 315200 P. R. China

**Keywords:** conformable bioelectronics, high‐fidelity biosensing, liquid metal nanofilms, low temperature deposition, ultra‐thin electrodes

## Abstract

Epidermal electrodes have demonstrated promising potential for high‐fidelity electrophysiological monitoring owing to their superior conformability. However, achieving conductive films that simultaneously possess ultrathin architectures and high conductivity to enable super conformal skin integration and enhanced signal‐to‐noise ratios under dynamic conditions remains a formidable challenge. Here, a ≈200 nm‐thick liquid metal (LM) conductive film and fabricate an epidermal bioelectrode is reported with a total thickness of merely 1.1 µm. The key innovation lies in a thermal‐low temperature synergistic deposition strategy that combines in situ substrate precooling with thermal evaporation, which fundamentally differs from conventional LM film techniques (e.g., printing or sputtering) by enabling precise nanoscale control over film continuity and uniformity. This approach effectively suppresses the dewetting behavior of LM through low temperature stabilization of nucleation sites, thereby accomplishing layer‐by‐layer deposition of defect‐free nano‐conductive films (minimum thickness: 19 nm). The resulting film exhibits exceptional conductivity (3×10⁶ S·m^−1^) without requiring mechanical activation. The fabricated epidermal electrode demonstrates outstanding skin conformability, ultralow interfacial impedance (8.5 kΩ at 1 kHz), and remarkable dynamic stability, enabling medical‐grade electromyographic signal monitoring during motion. These advances highlight its significant potential for wearable bioelectronic applications.

## Graphical abstract

A low‐temperature deposition strategy is proposed to fabricate 19 nm‐thick liquid metal nanofilms (conductivity: 3 × 10⁶ S m^−1^) for developing ultra‐thin, high‐conductivity epidermal electrodes. The 1 µm‐thick composite achieves 8.5 kΩ skin contact impedance and 26 dB EMG SNR, enabling robust dynamic electromyography during movement. Critically, this low‐temperature approach overcomes long‐standing manufacturing challenges in LM‐based ultrathin flexible bioelectronics.

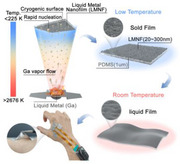

## 1 Introduction

Physiological electrical signals, including electrocardiogram (ECG),^[^ ^1^, ^2^, ^3^ ^]^ electroencephalogram (EEG),^[^ ^4^, ^5^ ^]^ and electromyogram (EMG),^[^ ^6^, ^7^, ^8^ ^]^ represent the fundamental electrophysiological manifestations of the human neuromuscular activity. Precise and stable acquisition of these signals is essential for clinical diagnosis, personalized health monitoring, human‐machine interaction (HMI), and brain‐machine interfaces (BMI).^[^ ^9^ ^]^ Key challenges in this domain include achieving a high signal‐to‐noise ratio (SNR), suppressing motion artifacts, and maintaining reliable signal capture under dynamic conditions.^[^ ^10^ ^]^ Epidermal bioelectrodes are critical in overcoming these challenges. According to the relation

(1) $$\;U_{\mathrm{conformal}}=U_{\mathrm{bending}}+U_{\mathrm{skin}}+U_{\mathrm{adhesion}}$$

where:

(2) $$\;U_{\mathrm{bending}}=\frac{1}{12\left(1-\upsilon^{2}\right)}\bar{E}h^{3}$$

($\bar{E}$ is Young's modulus, *ℎ* is the plate thickness, and *ν* is Poisson's ratio), reducing the thickness and elastic modulus effectively lowers the interfacial contact energy, thereby enhancing conformability between the electrode and skin.^[^ ^11^ ^]^ Thus, an ideal epidermal electrode should possess the following attributes: i) High electrical conductivity (>10⁶ S m^−1^) to suppress electromagnetic interference and enhance SNR;^[^ ^12^ ^]^ ii) An ultrathin structure (thickness < 1 µm) to minimize interfacial energy with skin, improving adhesion, reducing contact impedance (<10 kΩ), and suppressing motion‐induced noise for superior signal fidelity; iii) Mechanical properties compatible with skin biomechanics, including low elastic modulus (≈1 MPa) and high stretchability (>60%),^[^ ^13^, ^14^ ^]^ to ensure stable signal acquisition during body movements.^[^ ^15^ ^]^

In recent years, epidermal electrodes have advanced significantly through the integration of advanced functional materials. Such as conductive polymers,^[^ ^16^, ^17^ ^]^ carbon‐based materials,^[^ ^18^, ^19^ ^]^ and metals^[^ ^20^, ^21^ ^]^ and innovative structural designs, demonstrating promising applications in physiological electrical signal monitoring. A representative example is the ultrathin electrode based on the conductive polymer PEDOT: PSS, which has a thickness below 1 µm, an electrical conductivity of ≈100 S m^−1^, and stretchability ≈10%. When composited with graphene, the ultrathin electrode's thickness is reduced to ≈100 nm, while stretchability and conductivity improve to 40% and 4.14×10^5^ S m^−1^, respectively. Metal‐based epidermal electrodes, such as the Au/PDMS composite (≈1.3 µm thick), offer higher conductivity (1.58 ×10^6^ S m^−1^).^[^ ^22^ ^]^ However, their relatively high modulus limits skin conformability and dynamic stability. Although recent advances have embedded metallic nanowires into PDMS to achieve a favorable combination of low modulus (1.486 MPa) and low sheet resistance (0.4 Ω sq^−1^), their stretchability (54%) remains limited for applications requiring large deformations.^[^ ^23^ ^]^ Thus, realizing ultrathin epidermal electrodes that combine high conductivity with low modulus remains a critical challenge.

LMs, such as gallium (Ga) and its alloys, has garnered significant attention in flexible electronics applications due to their exceptional combination of metallic conductivity, fluidity, and biocompatibility.^[^ ^24^, ^25^, ^26^, ^27^, ^28^ ^]^ However, their inherently high surface tension (≈700 mN m^−1^, approximately ten times that of water) and rapid surface oxidation present fundamental challenges in fabricating continuous and ultrathin conductive films, particularly at the nanometer‐scale thickness.^[^ ^29^ ^]^ This has motivated diverse strategies ranging from surface energy patterning (e.g., using anti‐metal coatings to guide LM movement)^[^ ^30^ ^]^ to the creation of composite materials and microfluidic structures. Current LM film fabrication techniques (such as primarily screen printing, spray coating, and physical vapor deposition (PVD)) each have limitations.^[^ ^31^, ^32^, ^33^ ^]^ Screen printing and spray coating typically require surface activation and yield films with thicknesses ranging from micrometers to millimeters, restricting their suitability for ultrathin bioelectrodes. While PVD offers the potential to produce thinner films, the high surface tension of LMs often results in discrete, non‐conductive spherical particles rather than uniform continuous films.^[^ ^33^ ^]^ Beyond these, more recent approaches have sought to circumvent these challenges by structuring LMs within other materials. These include formulating LM‐polymer composites (e.g., with Ecoflex or gelatin) to create stretchable conductors and sensors,^[^ ^34^ ^]^ or confining LMs within microfluidic channels to pre‐define conductive pathways, where channel geometry itself becomes a key performance parameter.^[^ ^35^, ^36^ ^]^ While effective for specific applications, these methods inherently result in composite structures or microscale confinement, which can compromise the direct metal‐skin contact crucial for low impedance in bioelectronics and do not yield pure, nanometer‐thin films. Approaches such as incorporating metal interlayers or indium coatings can improve LMs wettability and facilitate the formation of submicron conductive films (<1 µm).^[^ ^37^, ^38^ ^]^ However, these methods often compromise electrode mechanical compliance and increase fabrication complexity. These constraints underscore the urgent need for innovative deposition techniques that enable direct formation of high‐quality, ultrathin LM films on elastic substrates without activation, while preserving the intrinsic properties of the metal. Such advancements are crucial for the development of flexible, biocompatible, and high‐performance bioelectrodes.

In this study, we report an innovative low‐temperature deposition technique for the fabrication of LM nanometer‐scale conductive films. By thermally evaporating LMs onto a precisely controlled low‐temperature substrate, rapid nucleation and crystallization are induced, shifting the growth mode from conventional island formation to a layer‐by‐layer process, enabling the creation of ultra‐thin conductive films that defy the inherent high surface tension of LMs, thereby achieving initial film conductivity. The resulting LM films exhibit a minimum thickness of 19 nm and an initial conductivity approaching 3 × 10⁶ S m^−1^. Leveraging this approach, nanometer‐thick LM films were subsequently deposited onto polydimethylsiloxane (PDMS) substrates, yielding ultrathin epidermal electrodes with a total thickness of ≈1 µm. These electrodes demonstrate excellent skin conformability, significantly reduced contact impedance (8.5 kΩ at 1 kHz), and maintain stable interfacial impedance under dynamic skin deformation. This enables high‐quality dynamic EMG signal acquisition with a signal‐to‐noise ratio up to 26 dB, highlighting their potential for complex gesture recognition.

In conclusion, our low‐temperature deposition strategy fundamentally circumvents these limitations by: 1) uppressing dewetting via in situ substrate precooling, which kinetically stabilizes LM nucleation and enables layer‐by‐layer growth (vs island formation in PVD). 2) Achieving 19 nm continuous films without activation layers, eliminating the trade‐off between conductivity and mechanical compliance. 3) Maintaining intrinsic LM properties (fluidity and stretchability) while providing initial conductivity (>3×10⁶ S m^−1^), surpassing existing LM films (<10⁵ S m^−1^ without post‐treatment). These advancements address the critical gap in conventional methods, where ultrathin LM films either lack continuity (PVD) or require compromises in flexibility (interlayer‐based approaches). This work provides novel solutions to critical challenges in LM film fabrication and establishes a robust technical foundation for developing high‐performance flexible bioelectrodes.

## 2 Results and Discussion

### 2.1 Design and Fabrication of the LMNF@PDMS

**Figure 1:**
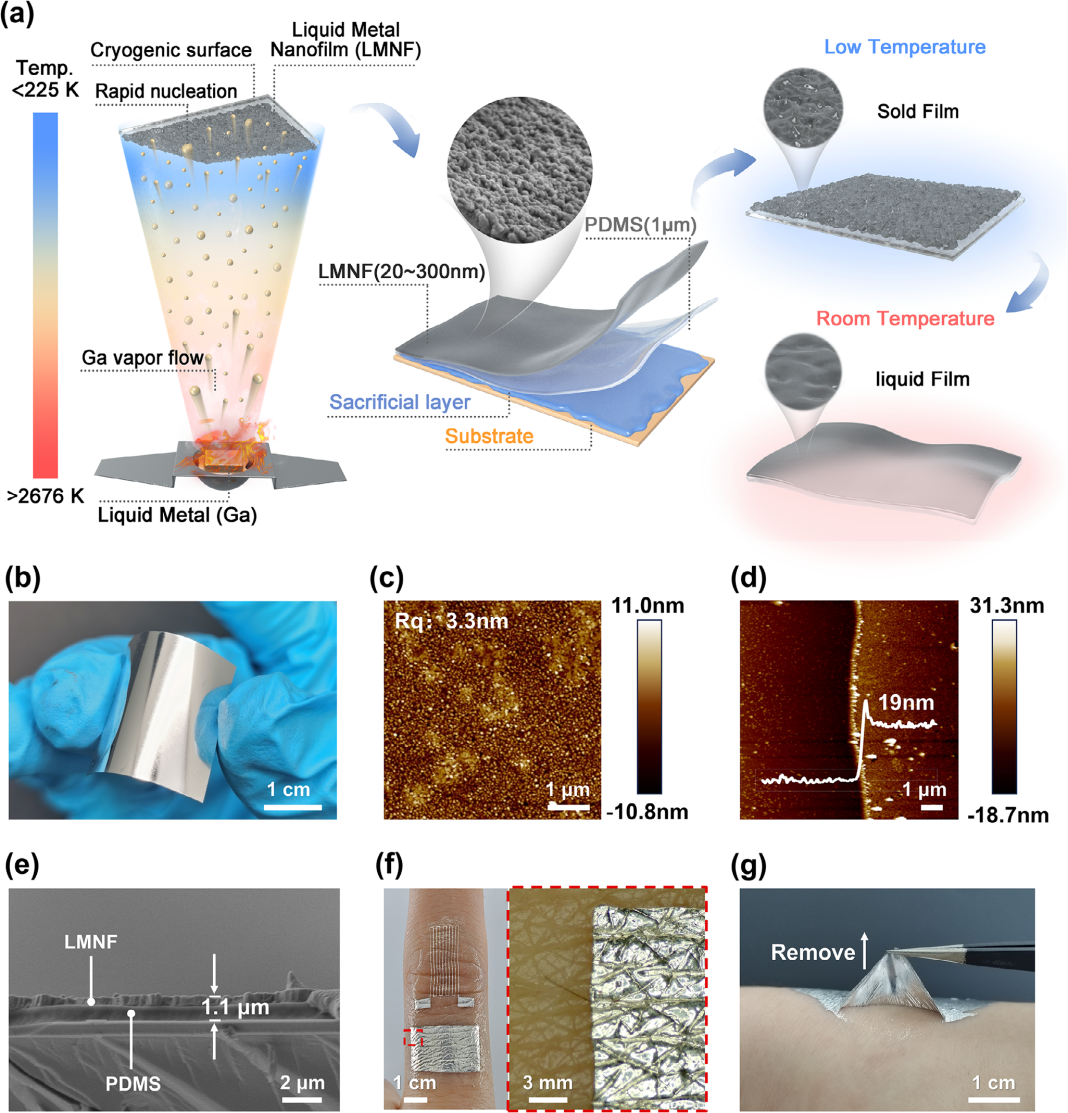
Stretchable self‐conformal LM conductive nanofilm. a) Schematic illustration of the fabrication process of the liquid metal nanofilm (LMNF) and its potential applications. b) Photograph of a large‐area LMNF film grown on a flexible substrate, exhibiting uniform and smooth surface morphology. c) AFM image displaying the surface roughness of the nanofilm. d) Atomic force microscopy (AFM) image showing the boundary of the LMNF and the height difference at the boundary. e) Cross‐sectional scanning electron microscopy (SEM) image of the LMNF/PDMS electrode attached to a Si substrate coated with dextran. f) Left: Photograph of the LMNF/PDMS electrode adhered to a finger. Right: Magnified view demonstrating the excellent conformability of the film to the skin. g) Peel‐off behavior of LMNF/PDMS electrodes from human skin.

To obtain nanoscale continuous LM films with initial conductivity, we designed and implemented a low‐temperature physical vapor deposition (CPVD) system that precisely controls the nucleation and growth dynamics of liquid metal films through a carefully engineered phase transition pathway. (**Figure** **1**a) Our innovative approach encompasses three key processes: i) Rapid nucleation of gas‐solid transition on the substrate surface at temperatures below 225 K, generating high‐density nanoclusters; ii) During the further continuous deposition process, control the solid‐state coalescence, presenting a layered growth to form continuous and dense solid‐state nano‐films; iii) Upon atmospheric exposure, a self‐limiting oxide film forms on the surface of the LM nanofilm. At a temperature of 304 K, a solid‐liquid transformation occurs, resulting in a stretchable and conductive LM nanofilm. The films obtained by this method have extremely high flatness and present a mirror‐like effect (specular reflectance >83.3% at 550 nm wavelength) (Figure 1b; Figure S1, Supporting Information). The characterization results of atomic force microscopy (AFM) indicate that the root mean square roughness (Rq) of the films is 3.3 nm over 5 × 5 µm^2^ scan areas (Figure 1c). The thickness of the thin films prepared by this method can be as low as a historic ≈19 nm, setting a new record as the thinnest stretchable LM conductors reported to date (Figure 1d). This combination of nano‐scale smoothness (Rq <5 nm) and nanoscale thickness (<20 nm) while maintaining bulk‐like conductivity (3.0 × 10⁶ S m^−1^) represents a significant breakthrough in gallium‐based flexible nanoelectronics fabrication. This deposition method exhibits broad substrate compatibility, allowing for the fabrication of conductive nanofilms on Si, polyimide (PI), PDMS, Ecoflex, and paper substrates (Figure S2, Supporting Information). Additionally, by integrating shadow masking techniques, we successfully patterned complex conductive circuits (Figure S3 and Movie S1, Supporting Information). To further enhance the resolution, we combined the approach with UV photolithography: spin‐coating a photoresist on the substrate, followed by exposure, development, and LM low‐temperature deposition, ultimately achieving micrometer‐scale precision patterning via lift‐off processing (Figure S4, Supporting Information).

Having established the nanofabrication paradigm for LM films, we now bridge this advancement to the development of ultrathin all‐elastic bioelectrodes in which both the conductive layer (LM) and substrate (PDMS) maintain nanoscale thickness and full elastomeric properties. Given its exceptional flexibility, stretchability, biocompatibility,^[^ ^39^ ^]^ and mechanical compatibility with biological tissues, PDMS was selected as the elastomeric substrate. By optimizing the dilution ratio with n‐hexane and spin‐coating (Figure S5, Supporting Information), we fabricated ultrathin PDMS substrates with thicknesses below 1 µm. Subsequently, ultrathin bioelectrodes were successfully produced using the low‐temperature deposition technique. Cross‐sectional scanning electron microscopy (SEM) imaging confirms a total electrode thickness of 1.1 µm (Figure 1e; Figure S6, Supporting Information). The intrinsic stretchability of both the substrate and conductive layer enables the freely suspended ultrathin electrode to withstand hydrostatic pressures exceeding 2900 times its own weight, corresponding to a tensile stress of ≈872.7 kPa (Figure S7 and Movie S2, Supporting Information). Moreover, owing to the ultrathin nature of the film, the electrode exhibits outstanding self‐conformability, enabling seamless adhesion to the skin surface (Figure 1f). The LMNF/PDMS electrode demonstrates epidermal‐compliant detachment capabilities, achieving interface separation without compromising skin integrity (Figure 1g).

### 2.2 Optimization of Process Parameters for Low Temperature Deposition

**Figure 2:**
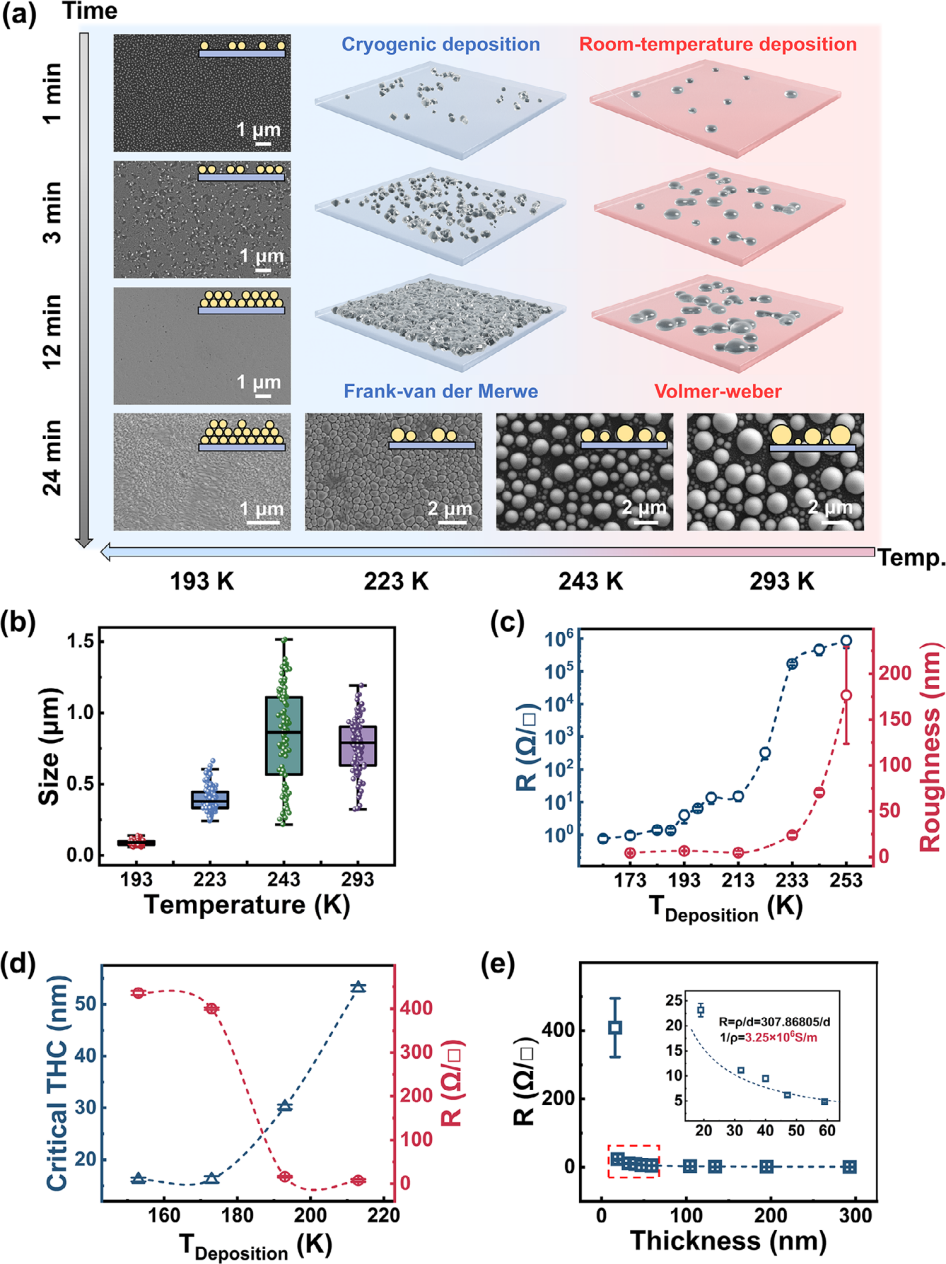
Parameter modulation in low‐temperature deposition of LM films and the mechanism of film growth. a) Microstructural morphology and growth patterns of the film under different substrate temperatures and deposition durations. b) Box‐plot analysis of LMNF nanoparticle size distributions deposited at different temperatures. c) Impact of deposition temperature on the ultimate thickness and sheet resistance of the film. d) Effect of varying evaporation rates on film thickness and surface roughness. e) Sheet resistance values of films with different thicknesses.

To systematically investigate the influence of substrate temperature and deposition time on the morphology and electrical properties of the films during low temperature deposition, we first deposited 0.7 g of Ga (corresponding to a fixed deposition time of 24 min under our experimental parameters) on substrates at four distinct temperatures: 293 K, 243 K, 223 K, and 193 K. This approach ensures that the total mass deposited remains constant across all temperature conditions, enabling a direct comparison of temperature‐dependent effects on film formation. **Figure** **2**a presents scanning electron microscopy (SEM) images of the films deposited at these temperatures. The experimental results reveal a significant reduction in particle size with decreasing substrate temperature. When the substrate temperature falls below 223 K, the film morphology transitions from discontinuous particulate structures to continuous flake‐like architectures. Further reduction in temperature leads to finer grain sizes and denser film structures (Figure 2b; Figure S8a, Supporting Information). Sheet‐resistance measurements (Figure 2c) demonstrate that films deposited at temperatures above 223 K exhibit high resistance, approximating an insulating state. In contrast, films deposited at 223 K begin to display conductive behavior, with the sheet resistance decreasing markedly as the substrate temperature is further reduced. The surface roughness characterization further confirms that lower deposition temperatures yield smoother films, with roughness values below 25 nm. This phenomenon can be attributed to the grain refinement theory: lower temperatures promote smaller grain sizes and denser film structures, thereby significantly reducing sheet resistance.

In addition, we investigated the critical thickness (the minimum film thickness required to form a continuous, electrically conductive LM nanofilm) of the conductive films at different deposition temperatures. As shown in Figure 2d, the critical thickness decreases with lower substrate temperatures, reaching an ultrathin value of ≈19 nm at 153 K. However, the sheet resistance of the thinnest continuous films achieved at each temperature decreased with increasing substrate temperature. This trend is governed by a fundamental trade‐off between percolation thickness and microstructural quality. At lower temperatures, severely limited adatom mobility enables the formation of a percolating conductive network at a reduced physical thickness; however, this comes at the cost of microstructural integrity. The resulting film is polycrystalline with a high density of grain boundaries and inherent defects leading to significantly higher sheet resistance. To elucidate the effect of deposition time on film morphology and performance, we fixed the substrate temperature at 193 K (selected for its low sheet resistance and surface roughness) and conducted deposition experiments on silicon (Si) substrates at a constant rate (0.35 KÅ s^−1^) for various durations (1, 3, 12, and 24 min). SEM images (Figure 2a) illustrate the evolution of film morphology with increasing deposition time: initially dispersed nanoparticles progressively interconnect to form a conductive network and eventually coalesce into a continuous film. Prolonged deposition leads to morphological stabilization without further significant changes. To simulate the deposition process, we systematically characterized the film thickness and surface roughness at different Ga deposition volumes (0.02, 0.05, 0.75, and 1 mL) (Figure S8b, Supporting Information). The results indicate that film thickness increases with deposition volume, while the surface roughness initially increases before decreasing. This trend may be explained by the following mechanism: during early deposition stages, atoms form a smooth monolayer via layer‐by‐layer growth, intermediate stages exhibit increased roughness owing to defect accumulation, and subsequent atomic diffusion fills voids, reducing roughness. Sheet‐resistance measurements (Figure 2e) reveal a pronounced decline with increasing film thickness. This trend is attributed to the evolution of film morphology during the initial growth stage. For films thinner than 100 nm, the slightly lower conductivity results from an incomplete coverage that disrupts the continuous electron transport pathways. As the film thickness increases beyond ≈50 nm, a fully dense, continuous conductive network is formed. The calculated conductivity for these thicker films (≥ 50 nm) reaches a high value of up to 3.25 × 10⁶ S m^−1^. This is evidenced by the excellent agreement between the experimental data points and the theoretical dashed curve in Figure 2e, which represents the expected sheet resistance for a film with a constant bulk conductivity of 3.25 × 10⁶ S m^−1^. This close alignment confirms that the electrical properties of our continuous films match the theoretical conductivity of bulk Ga. From a thermodynamic perspective, pre‐cooling the substrate to temperatures below a critical threshold prior to deposition fundamentally alters the growth mode of LMs from island (Volmer‐Weber) to layer‐by‐layer (Frank‐van der Merwe) growth (Figure 2a), in contrast to conventional thermal evaporation at ambient temperatures. This transition is attributed to increased undercooling at lower temperatures, which reduces the critical free energy for nucleation and decreases the critical nucleus radius, thereby promoting the formation of fine‐grained, continuous films. A detailed thermodynamic derivation is provided in the Supporting Information.

Kinetic analysis reveals that reduced substrate temperatures effectively suppress atomic and small cluster diffusion, “freezing” the fine‐grained microstructure. Deposited atoms tend to remain at their initial positions, while particle bombardment effects inhibit 3D island formation. Before the nuclei can diffuse and coalesce, newly arriving atoms cover the existing nuclei, resulting in ultrathin films with both fine grains and smooth surfaces. This low‐temperature deposition mechanism provides theoretical support for the fabrication of high‐performance conductive nanofilms.

Complementary TEM and XRD characterizations confirm the presence of solid‐phase domains in as‐deposited films. Crucially, these solid phases can be eliminated through a tailored thermal annealing process, thereby yielding pure liquid‐phase Ga nanofilms that remain in a stable supercooled state at room temperature (Figure S9, Supporting Information). This transition is consistent with the size and thermal‐history‐dependent suppression of covalent bond formation, which favors the liquid phase over the crystalline α‐Ga.^[^ ^40^ ^]^ The liquid state provides the nanofilms with controlled flowability. Cross‐sectional TEM combined with XPS analysis reveals a native oxide layer (≈2 nm Ga_2_O_3_) that enhances the mechanical stability of the film under strain while maintaining electrical conductivity (Figure S10, Supporting Information). Notably, compared to brush‐coated LM traces that exhibit fusion under stretching and pattern degradation upon folding, the low‐temperature deposited films maintain structural integrity and pattern fidelity even after severe mechanical deformation (Figure S11, Supporting Information). For enhanced stability during dynamic testing, we employ a ≈30 µm PDMS encapsulation layer (1:10 base: curing agent ratio) that conformally coats the films without compromising their stretchability. This stabilization approach differs fundamentally from conventional brush‐coated LM traces, which require complete thick encapsulation to prevent leakage. The PDMS encapsulation primarily serves to enhance electrical stability during stretching tests rather than preventing leakage, as the native oxide layer already provides sufficient containment for normal operation.

### 2.3 Mechanical, Electrical, and Permeability Analysis of LMNF/PDMS Ultrathin Electrodes

**Figure 3:**
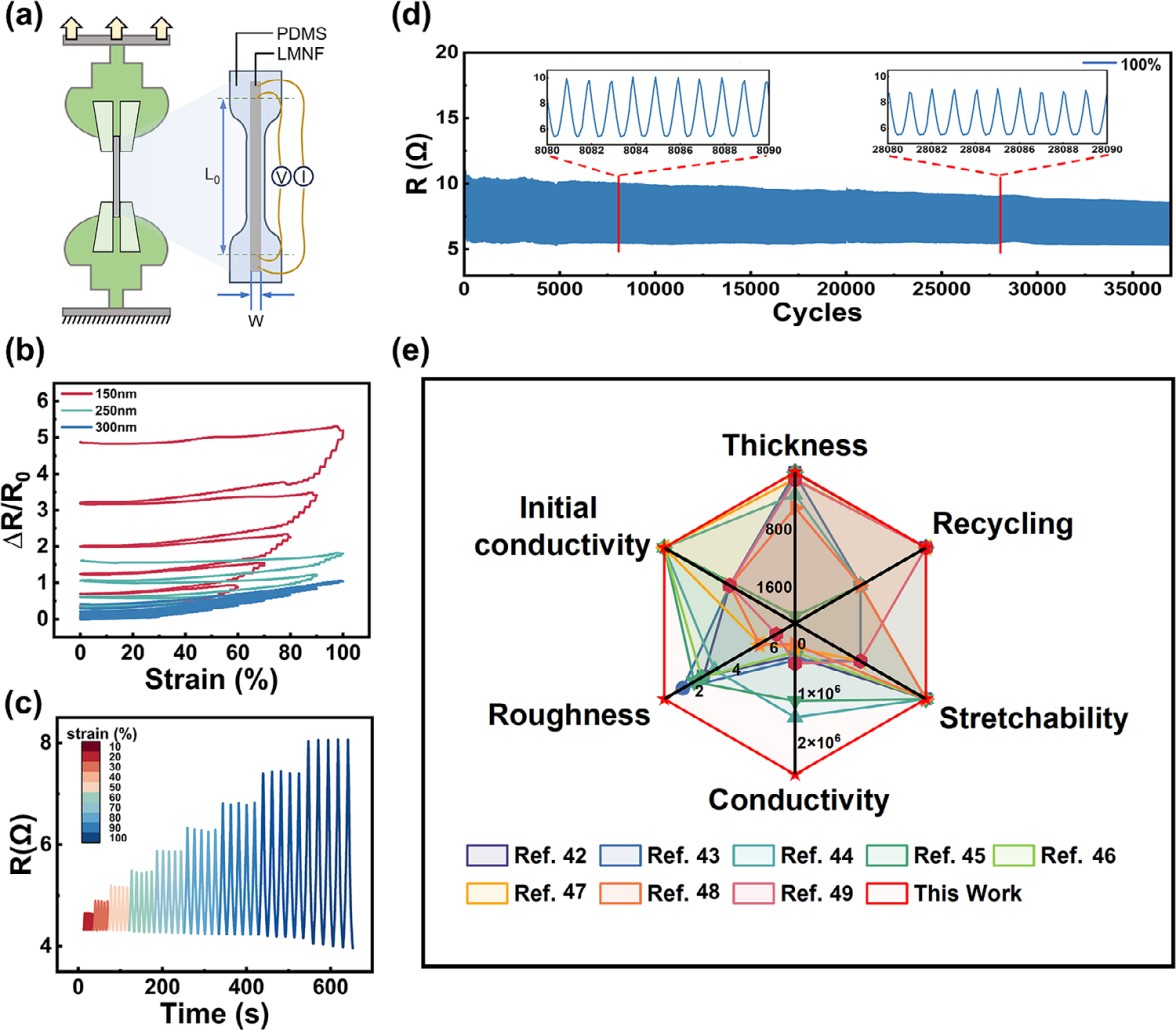
Multifunctional characterization of mechano‐electrical properties in LMNF. a) The sample is clamped by the mechanical stages and connected to a current source and a nanovoltmeter using a four‐terminal (Kelvin) configuration. The right shows a magnified view of the electrical connection scheme. b) Influence of liquid metal film thickness (150, 250, 300 nm) on electrical performance under tensile strain. c) Resistance variation of a 300 nm liquid metal film under different strains. d) Resistance changes of a 300 nm liquid metal film during 37000 cycles of 100% strain. e) Performance comparison between the LMNF and previously reported stretchable liquid metal films.

We conducted a systematic electromechanical characterization of the fabricated nanofilms to evaluate their thickness‐dependent performances using a four‐terminal method for electrical measurement coupled with a mechanical stretcher for strain application (**Figure** **3**a). Three LMNFs with varying thicknesses (150, 250, and 300 nm) were subjected to progressive tensile strain testing. Remarkably, all specimens demonstrated exceptional electromechanical stability, with a relative resistance change (*ΔR/R_0_*) at zero strain exhibiting distinct thickness dependence. Notably, the 300 nm‐thick LMNF maintained outstanding conductivity even after 100% strain, showing only a minimal 6% increase in baseline resistance (Figure 3b). Comparative analysis with 300 nm‐thick Au films revealed the superior stretchability of Ga‐based nanofilms: while Au films exhibited a dramatic *ΔR/R_0_* of 19.25 at merely 4.8% strain, the Ga films maintained a stable *ΔR/R_0_* of 2.31, even under 190% strain, representing an 8.3‐fold improvement in strain stability (Figure S12a, Supporting Information). It exceeds the stability of bulk liquid metal, which can be attributed to its unique polycrystalline microstructure with inherent oxide boundaries. XPS depth profiling (Figure S13, Supporting Information) confirms that the film interior, while predominantly metallic, retains traces of oxide. This suggests that a native oxide layer forms on the individual nanoparticles during the low‐temperature deposition process and persists at the grain boundaries after coalescence. Critically, these intergranular oxide layers have a thickness below 1 nm, which is within the characteristic length scale for quantum mechanical tunneling to occur efficiently.^[^ ^41^ ^]^ Consequently, electrical conduction is governed by electron tunneling across these ubiquitous, thin oxide barriers. Under strain, the established percolation network through these grain boundaries remains largely intact, effectively averaging out the resistance change and resulting in the observed minimal ΔR/R_0_. This mechanism contrasts with bulk liquid metal, where strain must disrupt a pristine, oxide‐free conductive path, leading to a larger resistance increase.

To investigate the dynamic response characteristics, we performed real‐time resistance monitoring on 300 nm‐thick LMNFs with 10% strain increments (*ε* = 10–100%). The films demonstrated highly reversible resistance changes during both loading and unloading cycles (Figure 3c), recovering to near‐initial resistance values upon strain release. This behavior confirms the exceptional elastic properties. Moreover, under repeated 80% stretching at varying frequencies, *ΔR/R_0_* remained consistently stable at 1.67±0.02, demonstrating remarkable frequency independence (Figure S12b, Supporting Information). Long‐term reliability was assessed by cyclic testing at 100% strain. The 300 nm‐thick LMNF exhibited extraordinary durability over 37000 cycles, maintaining a stable electromechanical performance throughout. The zero‐strain resistance showed minimal variation (|*ΔR/R_0_* | < 0.25) with only negligible signal attenuation (Figure 3d), confirming the exceptional robustness of our nanofilm design.

Compared to state‐of‐the‐art LM‐based electrodes, our LMNFs present several groundbreaking advantages (Figure 3e): 1) precisely tunable nanoscale thickness; 2) intrinsic conductivity without post‐treatment activation; 3) ultrasmooth surfaces (nanoscale roughness); 4) near‐bulk intrinsic conductivity (3 × 10⁶ S m^−1^); 5) superior stretchability; and 6) full recyclability. These combined properties represent a significant advancement over previous technologies in stretchable conductive materials.^[^ ^42^, ^43^, ^44^, ^45^, ^46^, ^47^, ^48^, ^49^ ^]^

### 2.4 Mechanical and Interfacial Characterization of Ultrathin LMNF/PDMS Electrodes

We systematically characterized the mechanical properties of both pristine PDMS substrates and PDMS/LM nanofilm composites. Remarkably, the Ga nanofilm‐deposited electrode maintains exceptional flexibility, with a Young's modulus (1.596 MPa) showing only a marginal increase compared to bare PDMS (1.11 MPa) (Figure S14, Supporting Information), closely matching the mechanical compliance of the human skin (1–2 MPa).

**Figure 4:**
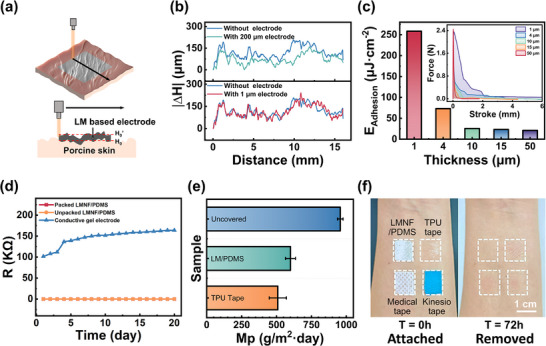
Electromechanical properties of ultra‐thin electrodes based on LM films. a) Schematic of thickness gauge scanning for porcine skin surface profilometry. b) Experimental surface profile comparison before and after electrode application (200 µm vs 1 µm thickness). c) Comparison of area‐specific adhesion energy for LMNF/PDMS electrode with different thicknesses on pig skin. d) Comparison of the time evolution of resistance between commercial hydrogel electrodes and LM electrodes. e) Comparison of the water vapor transmission rate (WVTR) for a bottle without a cover, a bottle covered with medical TPU bandages. f) Photographs illustrating the comparative skin irritation responses on the forearm of a volunteer before and after the application of LMNF/PDMS, medical TPU tape, medical adhesive tape, and sports tape.

To quantify skin conformability, we used laser profilometry to scan porcine skin surfaces before and after electrode attachment (**Figure** **4**a). Comparative analysis between 200 µm‐thick electrodes and our 1 µm ultrathin counterparts revealed a significantly enhanced conformal energy for the latter (Figure 4b). Step‐profiler measurements on silicon substrates with periodic microstructures demonstrated that for recessed features, the electrode‐substrate gap increases with the aspect ratio, while for protruding features, the gap decreases with the line width (Figure S15, Supporting Information). Critically, within the typical skin roughness range (1–10 µm), the maximum interfacial gap remains below 20 µm, which is crucial for high‐fidelity biosignal acquisition.

Adhesion performance was evaluated through peel‐off tests using porcine skin as the target surface. Universal tensile testing at a withdrawal speed of 10 mm min^−1^ revealed thickness‐dependent adhesion characteristics (Figure S16 and Movie S3, Supporting Information). The 1.1 µm‐thick LMNF/PDMS electrode exhibited optimal interfacial properties with an adhesion energy of 258.35 µJ cm^−2^ and adhesion length of 2.09 mm (Figure 4c), perfectly balancing the dual requirements of stable signal acquisition and non‐traumatic removal. This significant enhancement of adhesion with decreasing total film thickness can be attributed to a fundamental principle in mechanics: the bending stiffness (*D*) of a thin plate scales with the cube of its thickness (*D* ∝ *h* ^3^, as shown in Equation 2). The ultrathin electrode possesses exceptionally low bending stiffness, allowing it to conform effortlessly to the microscopic roughness and macroscopic curvatures of the skin surface with minimal elastic energy penalty. This superior conformability dramatically increases the effective contact area and facilitates more efficient dissipation of adhesion energy during the peeling process. In contrast, a thicker film is much stiffer and resists conforming to the skin; it bridges over skin valleys, resulting in a smaller real contact area and thus lower measured adhesion energy.

Long‐term stability assessments demonstrated superior performance over commercial electrodes (Figure 4d), with the LMNF/PDMS system maintaining consistent impedance characteristics essential for prolonged EMG monitoring. Notably, unlike hydrogel electrodes, which suffer from dehydration‐induced performance degradation over extended periods, our dry electrode system eliminates this fundamental failure mode. To quantitatively evaluate the stability under sweat exposure (a key challenge for long‐term wearable applications) we immersed the electrodes in artificial sweat. The results (Figure S17a, Supporting Information) confirmed functional stability (< 20 kΩ at 1000 Hz) over 1–2 days, which suffices for most practical scenarios. However, prolonged immersion led to a significant impedance increase, correlated with accelerated surface oxidation (Figure S17c–f, Supporting Information) and minimal Ga^3^⁺ release (78.5 µg cm^−^^2^ day^−1^, well below safety thresholds) (Figure S17b, Supporting Information) as characterized by XPS and ICP‐OES. This mechanistic understanding clarifies that during prolonged use in real‐world scenarios (e.g., multiple days of continuous wear), challenges such as sweat accumulation‐induced oxidation of the LM surface and potential weakening of skin adhesion may arise. These factors represent important avenues for future investigation, particularly through the development of advanced encapsulation strategies or surface modification techniques to enhance operational longevity. Furthermore, moisture permeability tests revealed a vapor transmission rate of 600.25 g m^−2^ day^−1^ (Figure 4e), exceeding medical‐grade TPU tapes (509.16 g m^−2^ day^−1^) and matching reported human perspiration rates (≈600 g m^−2^ day^−1^).^[^ ^37^ ^]^ This exceptional breathability prevents skin irritation during extended wear while maintaining electrical performance. Critical for clinical translation, we evaluated long‐term biocompatibility through 72‐h forearm wear tests comparing four materials: 1) LMNF/PDMS electrodes, 2) thermoplastic polyurethane (TPU) tapes, 3) medical adhesives, and 4) kinesiology tapes. While conventional materials induced erythema due to either poor breathability (TPU: 509 g m^−2^ day^−1^) or adhesive irritation, our electrodes caused no observable skin irritation (Figure 4f), attributable to their optimized vapor permeability (600.25 g m^−2^ day^−1^) and controlled adhesion energy (258.35 µJ cm^−2^).

### 2.5 On‐Skin Electrodes and Electrophysiological Monitoring

To demonstrate the practical utility of LMNF/PDMS ultrathin electrodes in cutaneous electrophysiology, we employed them for high‐fidelity EMG signal acquisition. The submicron thickness (1 µm) and skin‐matched modulus (1.596 MPa) enable seamless conformal contact with the epidermis (Figure S18, Supporting Information). Hydrophobic PDMS substrates can serve both as conformal electrode carriers and as inherent encapsulation layers. When LMs is laminated onto the skin and a seamless interface contact is established, PDMS forms a hydrophobic barrier to prevent liquid entry. This enables the electrode to have excellent water resistance and maintain signal integrity even during water exposure (Figure S19, Supporting Information).

**Figure 5:**
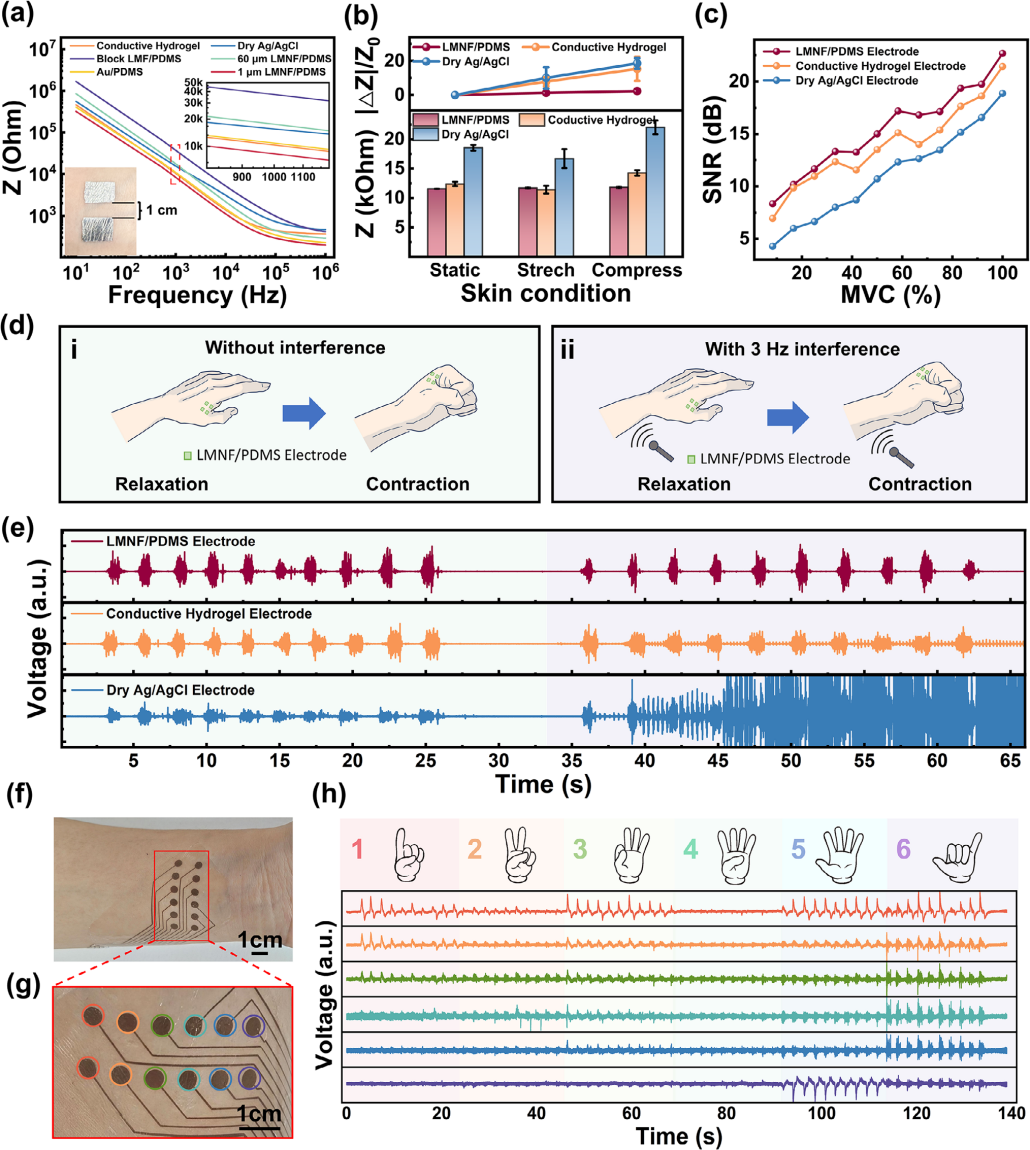
LMNF/PDMS ultra‐thin electrode: biocompatibility, impedance characteristics, and EMG monitoring. a) Skin contact impedance analysis of commercial dry electrodes, conductive hydrogel electrodes, the ultra‐thin LMNF/PDMS electrode, and Au/PDMS electrodes of the same thickness. b) Comparison of the skin‐contact impedance at 1 kHz among commercial dry electrodes, commercial conductive hydrogel electrodes, and the ultra‐thin LMNF/PDMS electrode, including impedance changes under compressed and stretched skin conditions. c) Signal‐to‐noise ratio (SNR) comparison among the ultra‐thin LMNF/PDMS electrode, commercial dry electrodes, and conductive hydrogel electrodes. d) Comparison of EMG signal acquisition at the first dorsal interosseous muscle: (i) Uninterrupted grip‐release cycles, (ii) 3 Hz mechanically perturbed motion. e) EMG signal acquisition from the first dorsal interosseous during grip‐release cycles under undisturbed and 3 Hz perturbed conditions. f) Optical image showing the conformal attachment of the array on the forearm. g) Magnified view of the six electrode pairs in the array. h) Simultaneously recorded six‐channel sEMG signals corresponding to the six hand gestures: (1) index finger pointing, (2) victory sign, (3) “OK” sign, (4) four‐finger extension, (5) “Shaka” sign, and (6) full hand open.

The skin‐electrode interface impedance is crucial for the detection of bioelectrical signals. We characterized the impedance at the electrode‐skin interface (**Figure** **5**a). Over a broad frequency range from 10 Hz to 10⁶ Hz, the contact impedance of the LMNF/PDMS electrode was significantly lower than that of conventional commercial Ag/AgCl dry electrodes, conductive hydrogel electrodes, and 1 µm‐thick Au/PDMS electrodes. This superior performance was consistent across multiple subjects (n = 3), with interfacial impedance at 1 kHz measuring 9.227±0.394 kΩ, demonstrating excellent reproducibility (Figure S20a, Supporting Information). The low impedance is quantitatively demonstrated in our EMG measurements, where the LMNF electrode consistently acquired signals with ≈38% higher amplitude than the commercial hydrogel electrode. This enhancement is directly attributable to its lower interfacial impedance (8.5 kΩ vs >10 kΩ for hydrogel at 1 kHz), which minimizes signal attenuation across the skin‐electrode interface. The overall thickness of the electrode was smaller, resulting in lower contact impedance with the skin (Figure S21, Supporting Information). Furthermore, we characterized the contact impedance of the electrode under static, stretched, and compressed states (Figure 5b). Results demonstrated that the LMNF/PDMS electrodes exhibited significantly smaller impedance variations under mechanical deformation (|△Z|/Z of only 1.26 in compression and 2.33 in stretching modes) compared to conventional conductive hydrogels (7.88 and 15.38, respectively) and dry electrodes (10.06 and 18.63, respectively). This superior electromechanical stability (Table S1, Supporting Information)^[^ ^11^, ^20^, ^44^, ^50^, ^51^, ^52^, ^53^, ^54^, ^55^, ^56^, ^57^, ^58^ ^]^ enables reliable electromyographic (EMG) signal acquisition during dynamic skin deformation. While spray‐coated liquid metal electrodes^[^ ^59^ ^]^ can achieve conformal contact, our approach further offers high‐precision patterning capability (readily achieving 50 µm feature size) and batch‐fabrication compatibility, ensuring consistent performance and scalable production. These advantages collectively facilitate high‐fidelity monitoring of muscular activities during motion.

For comparison, we simultaneously recorded the baseline noise under static and shaking conditions, as well as EMG signals during grip strength tests using commercial Ag/AgCl dry electrodes, conductive hydrogel electrodes, and LMNF/PDMS electrodes (Figures S22 and S23, Supporting Information). After attaching the electrodes to the forearm muscles, the grip strength was gradually increased from 1 to 12 kg. The results showed that the EMG signal amplitude increased with grip strength, and the signals recorded by the LMNF/PDMS electrode were consistently stronger than those from commercial electrodes. The signal‐to‐noise ratio (SNR) of the EMG signals improved with increasing grip strength, with the LMNF/PDMS electrode consistently outperforming the commercial electrodes (Figure 5c). At 12 kg grip strength, the SNR increased from 21.44 dB (commercial dry electrode) and 18.87 dB (conductive hydrogel electrode) to 22.68 dB (LMNF/PDMS electrode). Furthermore, tests across multiple subjects (n = 3) demonstrated reproducible performance, showing an SNR of 14.35±0.958 dB at a 6 kg grip force and underscoring the electrode's generalizability (Figure S20b, Supporting Information). Moreover, when detecting weaker grip forces, the LMNF/PDMS electrode could capture EMG signals as low as 0.8 kg (Figure S24, Supporting Information), whereas the commercial Ag/AgCl dry electrode failed to distinguish the signal from noise.

Additionally, the electrode demonstrated highly reproducible performance during repetitive gripping motions. The power distribution of the electrode remained stable across different frequencies, and the root mean square (RMS) amplitude of the acquired EMG signals showed a low coefficient of variation (0.08594, n = 6) (Figure S25, Supporting Information). Furthermore, as shown in Figure 5d, we collected EMG signals from the first dorsal interosseous (FDI) muscle under both static conditions and external tapping‐induced interference (Figure 5e; Movie S4, Supporting Information). In the static state, the LMNF electrode exhibited stronger EMG signals and lower baseline noise compared to commercial dry electrodes (Figure S26a, Supporting Information). When mechanical disturbances were introduced by tapping the arm to simulate motion artifacts, the LMNF electrode maintained a significantly lower baseline noise and clearer EMG signals than both the commercial dry and conductive hydrogel electrodes (Figure S26b, Supporting Information). During the electromyogram acquisition process with different movement amplitudes (small, medium, large) and contraction frequencies (1, 2, 4, and 7 Hz), the LMNF/PDMS electrode demonstrated superior performance. Compared with commercial dry electrodes and hydrogels, it had lower baseline noise under large movements and a faster response speed under high‐frequency contractions (Movie S5, Supporting Information). The conformal interface adhesion of the electrode (Movie S6, Supporting Information) and its inherent noise suppression characteristics jointly contribute to its clinical‐grade EMG monitoring capability, which is used for precise force estimation applications. To further evaluate performance under physiologically relevant conditions, we tested the electrode during large‐amplitude motions and prolonged exercise that induced sweating (Figure S27, Supporting Information). The electrode successfully captured stable EMG signals during running at various speeds, confirming its robustness against dynamic deformations. However, under prolonged, intense activity leading to profuse sweating, a gradual increase in baseline noise was observed, a challenge common to many dry electrodes due to the unstable electrolyte layer formed by sweat.

These results underscore a key advancement of our technology: it establishes a new class of dry electrode that achieves signal fidelity comparable to the clinical gold standard (hydrogel) in static conditions, while offering transformative stability in dynamic, real‐world applications under moderate sweat conditions. This transformative stability, enabled by the electrode's superior conformability, ensures reliable EMG acquisition in real‐world scenarios. Future iterations will focus on optimizing the interface to enhance performance under extreme perspiration.

The LMNF/PDMS electrodes were further used for robust dynamic EMG monitoring of six standard diving movements. The results showed that the differences in mean power frequency (MPF) and median frequency (MDF) between postures were statistically significant (Figure S28, Supporting Information). This frequency‐domain discriminability, maintained without signal degradation during skin deformation, highlights their unique advantage for machine learning‐based gesture classification systems, outperforming conventional rigid electrodes that suffer from motion‐induced spectral artifacts. To further showcase the versatility of our deposition technique for fabricating functional devices, we created a patterned six‐channel electrode array and applied it to multi‐channel sEMG recognition. The array was conformally attached to the forearm, where it acquired muscle activation signals simultaneously from six spatially distinct locations (Figure 5f,h). The electrode array was designed to distinguish between six distinct hand gestures: 1) index finger pointing, 2) victory sign, 3) “OK” sign, 4) four‐finger extension, 5) full hand open, and 6) “Shaka” sign. The resulting sEMG signals (Figure 5g; Figure S29, Supporting Information) exhibited characteristic patterns for each gesture. To quantitatively evaluate the classification performance, we employed a multi‐level convolutional neural network (MLCNN) for offline recognition (Figure S30a, Supporting Information). The model demonstrated robust performance with high overall accuracy (97.50±2.207% across n = 3 subjects), effectively distinguishing all six gestures by leveraging the spatiotemporal features (e.g., amplitude, timing, and spatial distribution) integrated across all six channels (Figures S30b and S31, Supporting Information). To assess practical robustness, we further evaluated the system's sensitivity to electrode placement errors. Translating the electrode array by 5 and 10 mm along the muscle fiber direction resulted in a quantifiable decrease in recognition accuracy to 91.1% and 89.81%, respectively. This highlights the importance of consistent placement for optimal performance and informs future work on enhancing alignment tolerance through algorithmic compensation or array design (Figure S32, Supporting Information). This result not only validates the high signal quality and spatial resolution of our patterned LM films but also underscores their significant potential for developing high‐information‐density biointerfaces and enabling refined human‐machine interaction.

## 3 Conclusion

We present a low temperature deposition technique for the direct fabrication of ultrathin LM conductive films, achieving thicknesses as minimal as 19 nm. These films demonstrate remarkable intrinsic conductivity, reaching up to 3 × 10⁶ S m^−1^, without necessitating mechanical activation or complex processing steps, due to a meticulously controlled layer‐by‐layer growth mechanism. Building upon this innovation, we developed an ultrathin epidermal electrode with a mere thickness of 1.1 µm, which demonstrates excellent air permeability (600.25 g m^−2^ day ^−1^). Meanwhile, thanks to the adaptive conformability of ultrathin LM electrodes, it can ensure close conformity to the skin and exhibits ultralow interfacial impedance (8.5 kΩ at 1 Hz). This facilitates the reliable acquisition of high‐fidelity EMG signals under dynamic conditions, achieving an SNR of 22.68 dB. The exceptional dynamic stability of the electrode enables precise responsiveness in complex gesture recognition, providing critical technical support for applications in intelligent prosthetic control, motion tracking, and human‐machine interfaces.

## 4 Experimental Section

### Fabrication of the LMNF

The LMNF was fabricated using a multifield PVD system under low‐temperature conditions. The LM films were deposited via thermal evaporation using a custom‐built multi‐field physical vapor deposition (PVD) system. This system was designed to apply and precisely control multiple external fields (including thermal, low temperature, and electrical) during the deposition process. For the specific experiments in this work, the key feature utilized was the precise temperature control of the substrate. This was achieved by a liquid helium‐cooled sample stage, which provides the base cooling power. The stage was integrated with a resistive heating element and a closed‐loop temperature feedback system, enabling accurate and stable temperature regulation from 15 K to 300 K. The substrate temperature was maintained at the desired setpoint (193, 223, 243, or 293 K) with a stability of ±1 K throughout the deposition process. The detailed schematic and photographs of the custom PVD setup are provided in Figure S33 (Supporting Information) for clarity. The substrate was placed on the sample stage inside the vacuum chamber of the PVD system. Liquid helium was employed to cool the sample stage, and Ga was used as the evaporation material. Prior to evaporation, the sample stage temperature was reduced to below 220 K and maintained for at least 1 h to ensure proper thermal stabilization. The evaporation process commenced with an evaporation current of 175 A, and the vacuum level was maintained below 3.0 × 10^−4^ Pa throughout the deposition. After a sufficient evaporation time, the evaporation current was terminated, and the substrate temperature was gradually increased back to room temperature at a rate of less than 100 K h^−1^ to prevent thermal stress. This process yielded a continuous and conductive LMNF.

### Fabrication of Ultrathin Electrodes (LMNF/PDMS)

The surface of the silicon (Si) substrate was pretreated using oxygen plasma (100 sccm, 150 W, 3 min). A 10 wt.% dextran solution was spin‐coated onto the pretreated substrate at 2000 rpm for 20 s, followed by sequential baking at 80 °C for 3 min and 180 °C for 30 min to obtain the pretreated substrate. Subsequently, a diluted precured solution consisting of 6 vol.% polydimethylsiloxane (PDMS, Sylgard 184, Base/Cross‐linker = 10:1, Dow Corning) in n‐hexane (Mw 86.18, Sigma‐Aldrich) was prepared. The solution was stirred at room temperature at 400 rpm for 1 h to ensure homogeneity. The diluted PDMS solution was then spin‐coated onto the pretreated substrate at 4000 rpm, followed by the deposition of a 1µm‐thick PDMS layer, which was thermally cured at 80 °C for 2 h to achieve complete cross‐linking. The substrate with the 1 µm‐thick PDMS layer was placed on the sample stage of a multifield PVD system. The LMNF conductive layer was fabricated using the low‐temperature deposition method. After the deposition, the sample was immersed in deionized water until the dextran layer was completely dissolved. Once the LMNF/PDMS film was fully released from the silicon substrate and floated on the water surface, it was carefully transferred to the skin surface for further application.

### Characterization of LMNF and LMNF/PDMS

The frontal and side‐view microstructures of the LMNF and LMNF/PDMS on the Si wafer were characterized by field emission scanning electron microscopy at an accelerating voltage of 4 kV (ZEISS, Sigma 200). The LMNF on Cu grid was observed under a high‐resolution transmission electron microscopy (HRTEM, Thermo Fisher, Talos F200x). The surface compositions of the LMNF were analyzed using X‐ray photoelectron spectroscopy (XPS, AXIS SUPRA, UK) with a reference of the C 1s peak at 284.6 eV. The phase composition of thin films was characterized by X‐ray diffraction (XRD, D8 DISCOVER, Germany) at Cu Kα radiation (wavelength λ = 0.15406 nm). The instrument had a tube voltage and current of 40 kV and 40 mA, respectively, with a scan step of 0.02° and an acquisition time of 0.3 s per step. The surface roughness of the test samples was studied using a surface profiler (DektakXT, Bruker, Germany) and a scanning probe microscope (Dimension ICON, Bruker, Germany) with a scanning range of 5 µm × 5 µm.

### Measurements of Electrical and Mechanical Properties of the Applied Strain

The mechanical properties were measured using a universal material testing machine (Instron 5943, USA), in which the loading rate was 50 mm min^−1^. The resistance change according to the stretching was measured using the DC source (Keithley 6221) and the nanovoltmeter (Agilent 34420A) by the four‐wires method. The sheet resistance of the LMNF was measured by a four‐probe resistivity tester (RTS‐9, Guangzhou 4Probes Tech Ltd.). Cyclic tensile strain was measured by a locally developed tensile fatigue machine.

### Peeling Force Measurement

For the adhesion‐separation measurement, the LMNF/PDMS ultrathin electrode was mounted on an acrylic frame with a hollowed‐out structure measuring 4 cm × 3 cm. The initial contact area between the LMNF/PDMS ultrathin electrode and porcine skin was set to 2.8 cm × 2.8 cm. The adhesive force was recorded using a universal tensile testing machine as the holder was gradually lifted at a speed of 10 mm min^−1^ until the ultrathin electrode was completely detached from the porcine skin. The adhesion energy was calculated using the following equation:

(3) $$E=\frac{\int_{0}^{x}\mathrm{ydL}}{S}$$

where *E, x, y, L*, and *S* represent the adhesion energy, separation stroke, separation force, stroke, and contact area, respectively.

### Evaluation of WVTR Property

Thirty‐three grams of deionized water were measured and transferred into glass bottles with an opening diameter of 12 mm. The bottles, each covered with different materials, were placed in a constant‐temperature cabinet maintained at 25 °C with a relative humidity of 20%. After 24 h, the weight loss of each bottle was recorded. The water vapor transmission rate (WVTR) was calculated using the following equation:

(4) $$\mathrm{MVTR}=\frac{m_{\mathrm{loss}}}{t\times S}\;$$

where *m_loss_*, *t*, and *S* are the water loss weight, time, and surface area, respectively.

### Impedance Measurement of the Electrode

Electrochemical impedance spectroscopy (EIS) measurements were performed by placing two electrodes with connectors on the skin at a distance of 1 cm from each other. One of the electrodes acted as the working electrode (cathode) and the other electrode acted as the reference/counter electrode (anode). Impedance measurements were performed on commercially available electrodes (2223H, 3 M), Ag/AgCl electrodes, and LMNF@PDMS electrodes. The impedance was measured using an impedance meter (IM 3570, HIOKI, Japan) and software (LCR Meter Sample Application) with a frequency range of 10 ≈10^6^ Hz. Impedance measurement was performed during the stretching and compressing of the electrode.

### EMG Recording

Delsys sEMG system (DELSYS Trigno lab, USA) was chosen to collect signals. The LNMF/PDMS electrodes were affixed to the left forearm and then connected to the Delsys sEMG system. The sample rate of the EMG recording was 1927 Hz. Signal processing of collected data using MATLAB for basic signal analysis (root mean square/spectrogram/fast Fourier transform). This study involves non‐invasive measurements with anonymous data collection, thus exempt from ethics committee approval.

### Data Preprocessing and Presentation

The raw electromyographic (EMG) signals were processed using MATLAB. The preprocessing pipeline included band‐pass filtering (20–450 Hz) to remove motion artifacts and high‐frequency noise, followed by a notch filter (50 Hz) to eliminate power line interference. All quantitative data were presented as mean ± standard deviation (SD) unless otherwise specified. The sample size (n) for each experiment was explicitly stated in the corresponding figure legend, representing the number of independent subjects or measurements.

### Sample Size

For the EMG signal acquisition and gesture recognition tasks, data were collected from n = 3 healthy adult subjects. Each gesture was repeated ten times per subject to constitute the training and testing datasets for the machine learning model. Four volunteers participated in this experiment. All of the volunteers gave written informed consent about the experimental procedure.

## Conflict of Interest

The authors declare no conflict of interest.

## Author Contributions

The authors hereby specify the individual contributions of each author to ensure transparency and accountability: S.L. performed in conceptualization, methodology, formal analysis, and writing – original draft. S.Y. performed in investigation, validation, writing – review and editing, software. J.L., F.X., Y.W.* performed in formal analysis, writing – review & editing, visualization. Q.Z., Z.H. performed in writing – review and editing, and visualization. J.S., C.S. performed in resources, software. Y.L.*, R.‐W.L.* performed in methodology, validation, supervision, writing – review & editing, project administration, and funding acquisition.

## Supporting information



Supporting Information

Supplemental Movie 1

Supplemental Movie 2

Supplemental Movie 3

Supplemental Movie 4

Supplemental Movie 5

Supplemental Movie 6

## Data Availability

The data that support the findings of this study are available in the supplementary material of this article.
