# Supplementary material for: Hundred‐Nanometer‐Thick Stretchable Liquid Metal Films for Ultra‐Conformal Bioelectrodes
Source: Adv Sci (Weinh). 2025 Oct 23;13(1):e12762. doi: 10.1002/advs.202512762 (PMC12767024; doi:10.1002/advs.202512762)
Supplement: Supplementary file 1 — Supporting Information [file ADVS-13-e12762-s005.docx]

Supporting Information

Hundred-nanometer-thick Stretchable Liquid Metal Films for Ultra-conformal Bioelectrodes

*Shiying Li, Shuai Yang, Jinyun Liu, Feng Xu, Yuanzhao Wu, Qi Zhang, Zidong He, Jie Shang, Yiwei Liu*, Run-Wei Li**

S. Li, S. Yang, J. Liu, F. Xu, Y. Wu*, Q, Zhang, Z He, Prof. J. Shang, Prof. C. Shi, Prof. Y. Liu*, Prof. R.-W. Li*

Ningbo Institute of Materials Technology and Engineering, Chinese Academy of Sciences, Ningbo, 315201, P. R. China.

wuyz@nitme.ac.cn (Prof. Y. Wu); liuyw@nimte.ac.cn (Prof. Y. Liu); runweili@nimte.ac.cn (Prof. R.-W. Li)

S. Li, S. Yang, J. Liu, F. Xu, Y. Wu*, Q, Zhang, Z He, Prof. J. Shang, Prof. Y. Liu*, Prof. R.-W. Li*

Center of Materials Science and Optoelectronics Engineering, University of Chinese Academy of Sciences, Beijing 100049, China

Prof. R.-W. Li*

Eastern Institute of Technology, Ningbo, 315200, China.

rwli@eitech.edu.cn


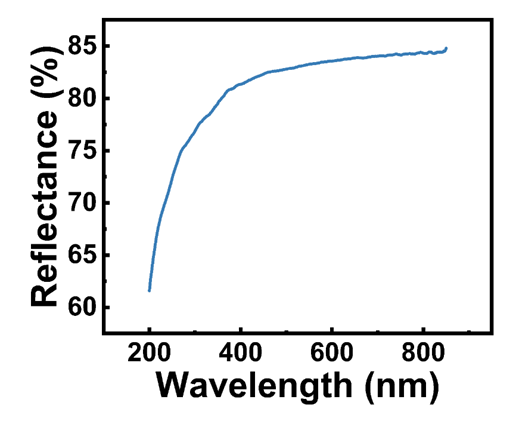


**Figure S1.** Wavelength-dependent reflectivity of 300 nm-thick LM Nanofilms.


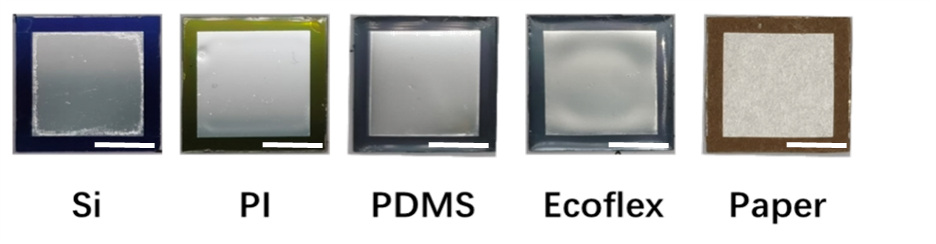


**Figure S2.** Digital images of LM films on various substrates. (scale bar of 1cm)


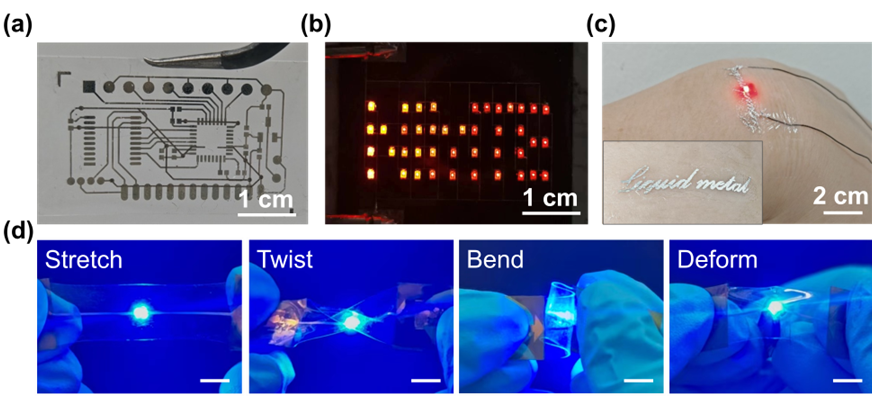


**Figure S3.** Gallium nanofilm interconnects for stretchable optoelectronic Systems. (a) Metal shadow mask-patterned LM circuits. (b) Digital image of encapsulated stretchable circuit with Ga-interconnected LEDs fabricated via low temperature deposition. (c) Conformable attachment of Ga-wired LEDs on a flexed wrist. (d) Mechanical robustness demonstration under multi-axis deformations: stretching (ε=80%), twisting (180°), bending (r=2 mm), and deform (scale bar of 1 cm).


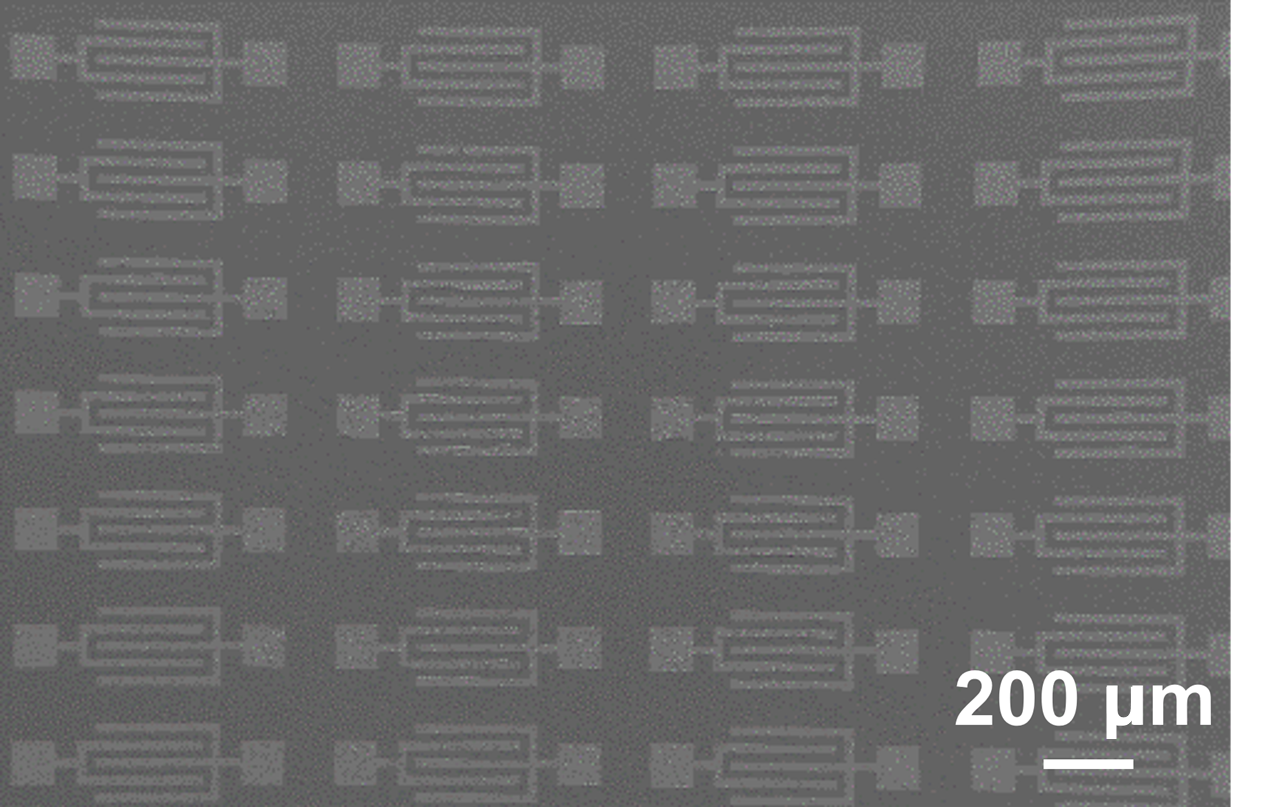


**Figure S4.** Lithography-fabricated LM interdigitated electrode array.

**
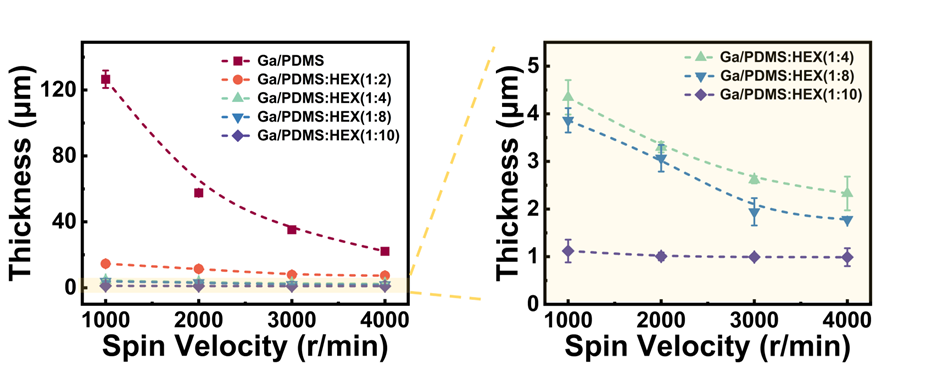
**

**Figure S5.** Thickness control of LM based bioelectrodes via hexane-modulated spin coating.

**
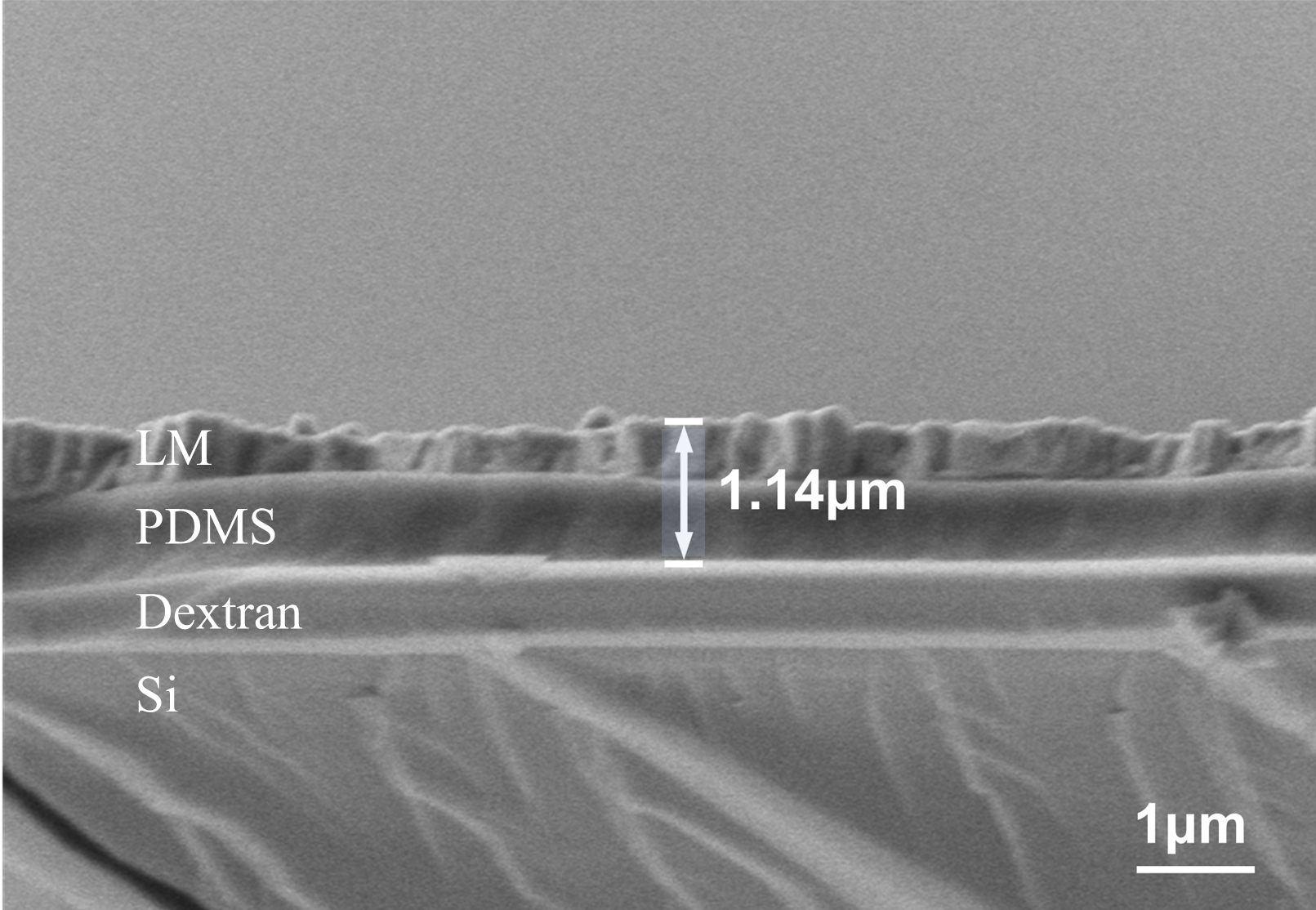
**

**Figure S6.** **Cross-sectional SEM characterization of the LM/PDMS layered structure.**


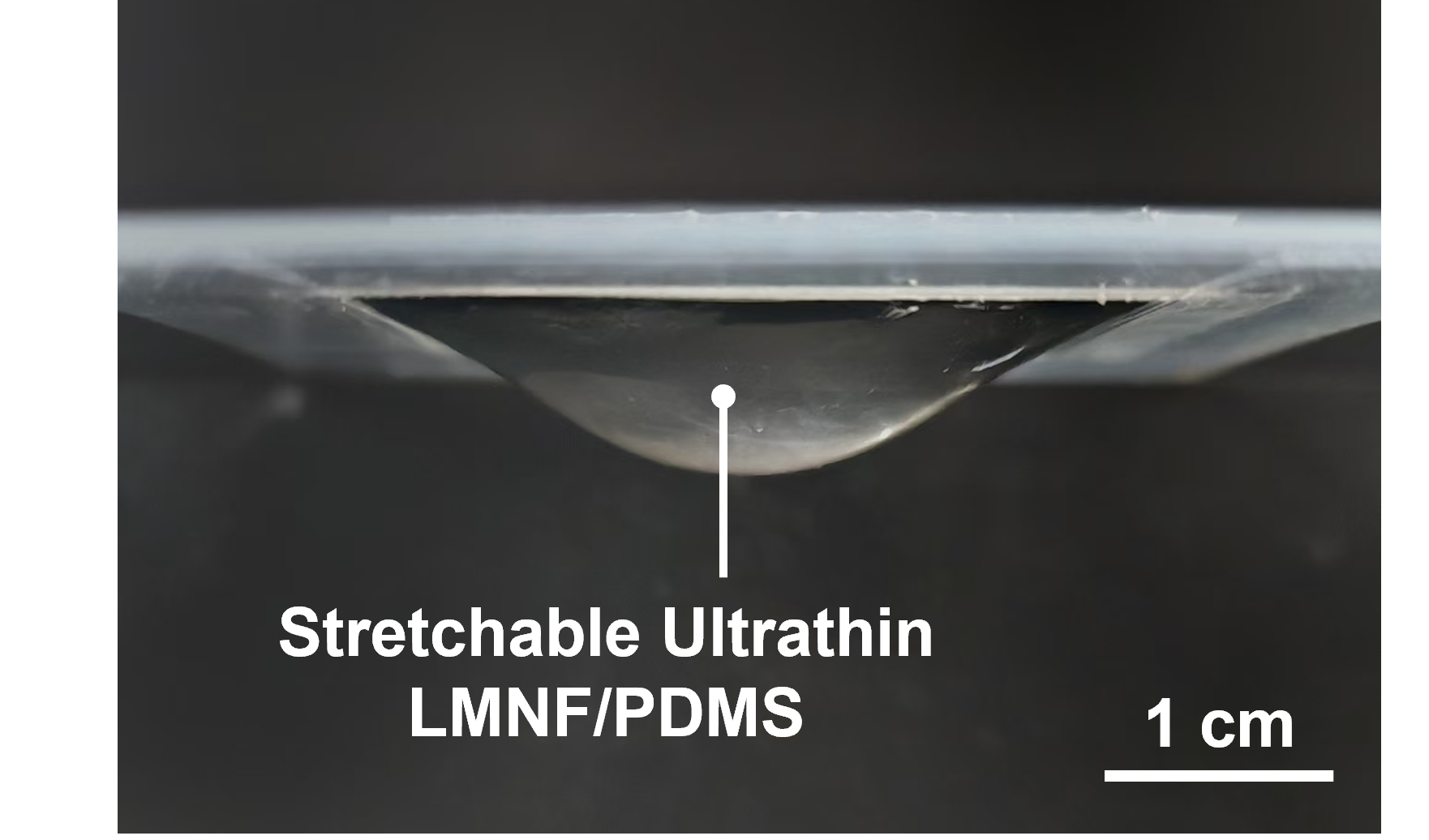


**Figure S7.** Photograph of a suspended ultra-thin LMNF/PDMS electrode holding a large amount of water.


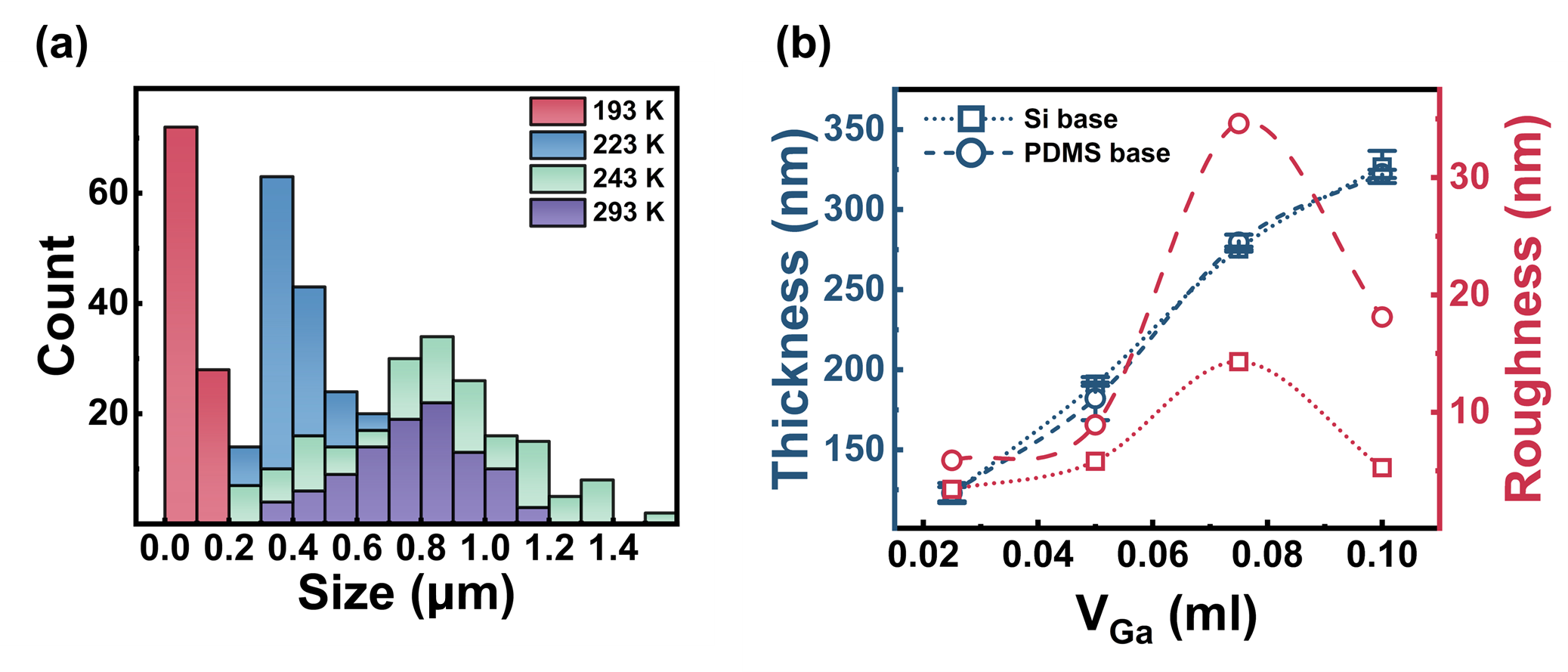


**Figure S8.** (a) Temperature-dependent particle size distribution histograms of LM nanoparticles. (b) Influence of deposition temperature on the resistance and surface roughness of films with identical thickness.


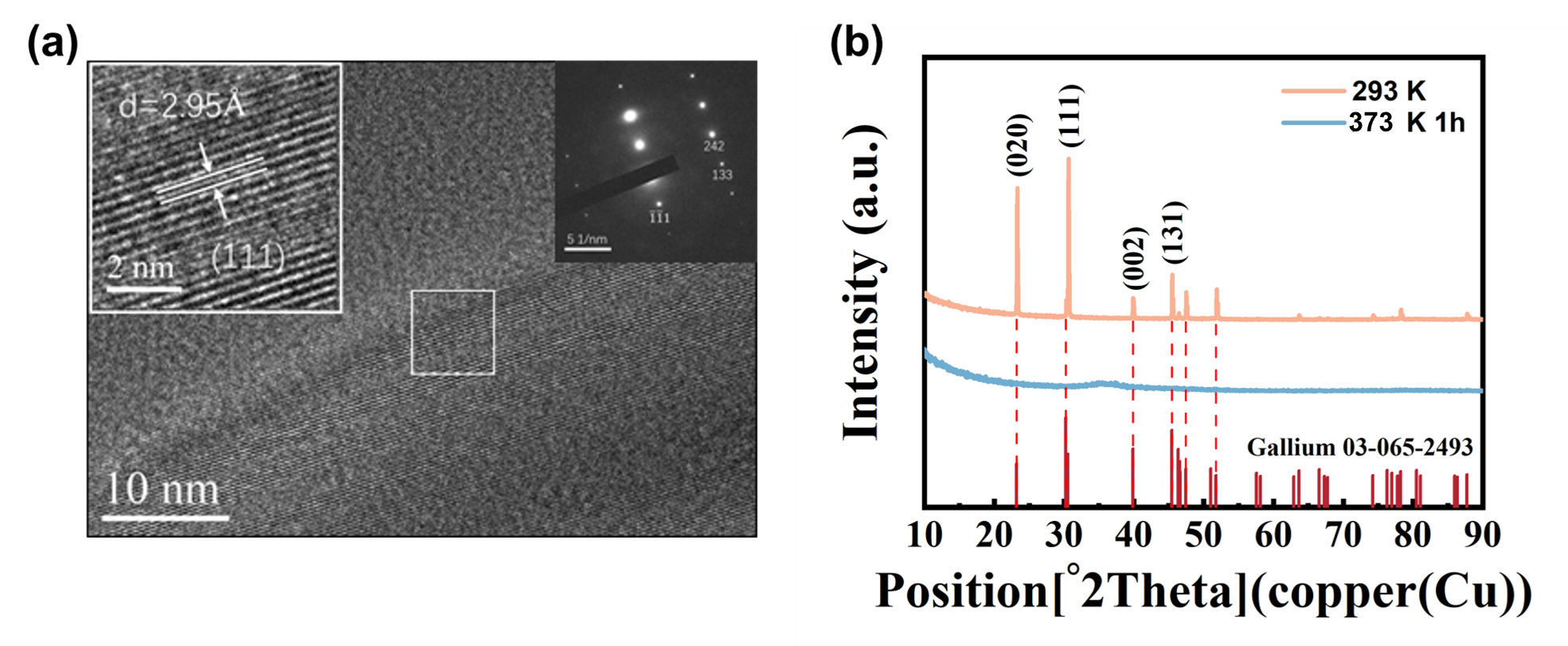


**Figure S9.** TEM and XRD characterization of Ga ultrathin films. (a) Low-magnification image showing continuous film morphology (Insert: High-resolution TEM revealing crystalline Ga domains ). (b) XRD analysis of Ga thin films before and after 100℃ annealing.


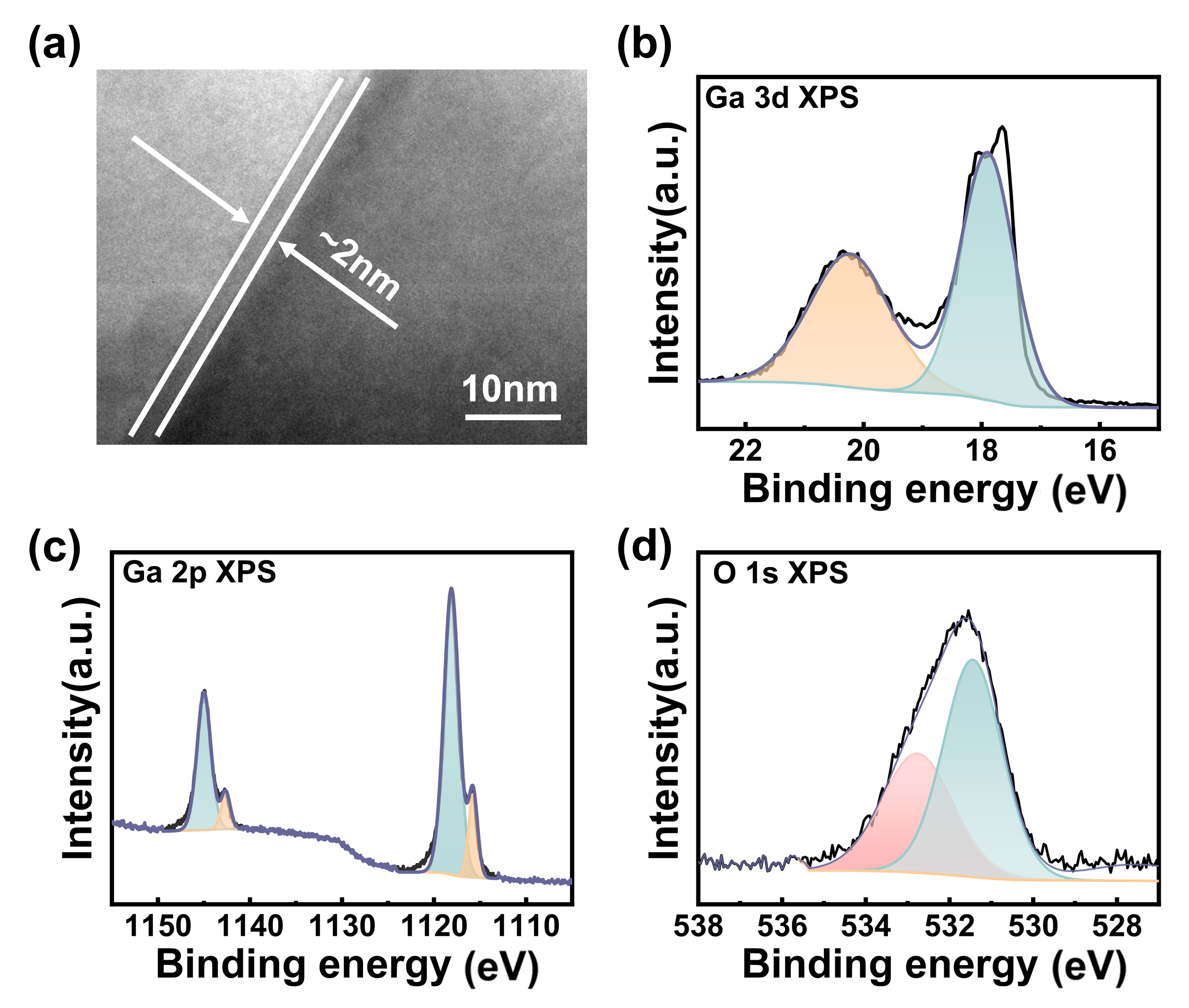


**Figure S10.** (a) TEM characterization revealing 2 nm-thick Ga₂O₃ interfacial layers. (b-d) XPS spectra of the gallium thin film: (b) Ga 3d, (c) Ga 2p, and (d) O 1s core-level regions, showing dominant oxidation states.


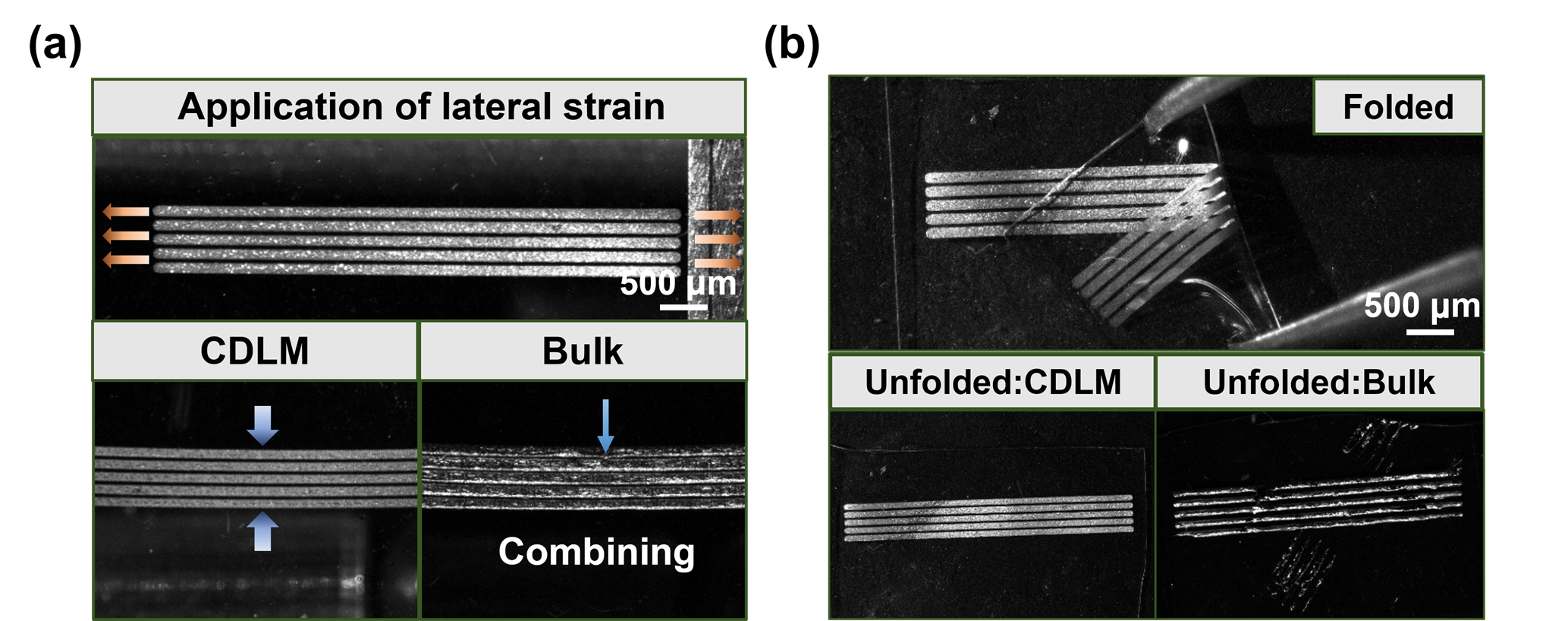


**Figure S11.** (a)Top: photograph of stretched CDLM. Bottom: images of CDLM (left) and bulk LM (right) after stretching. (b)Top: photograph of folded CDLM (r=0.5mm). Bottom: images after unfolding of folded CDLM (left) and bulk LM (right).


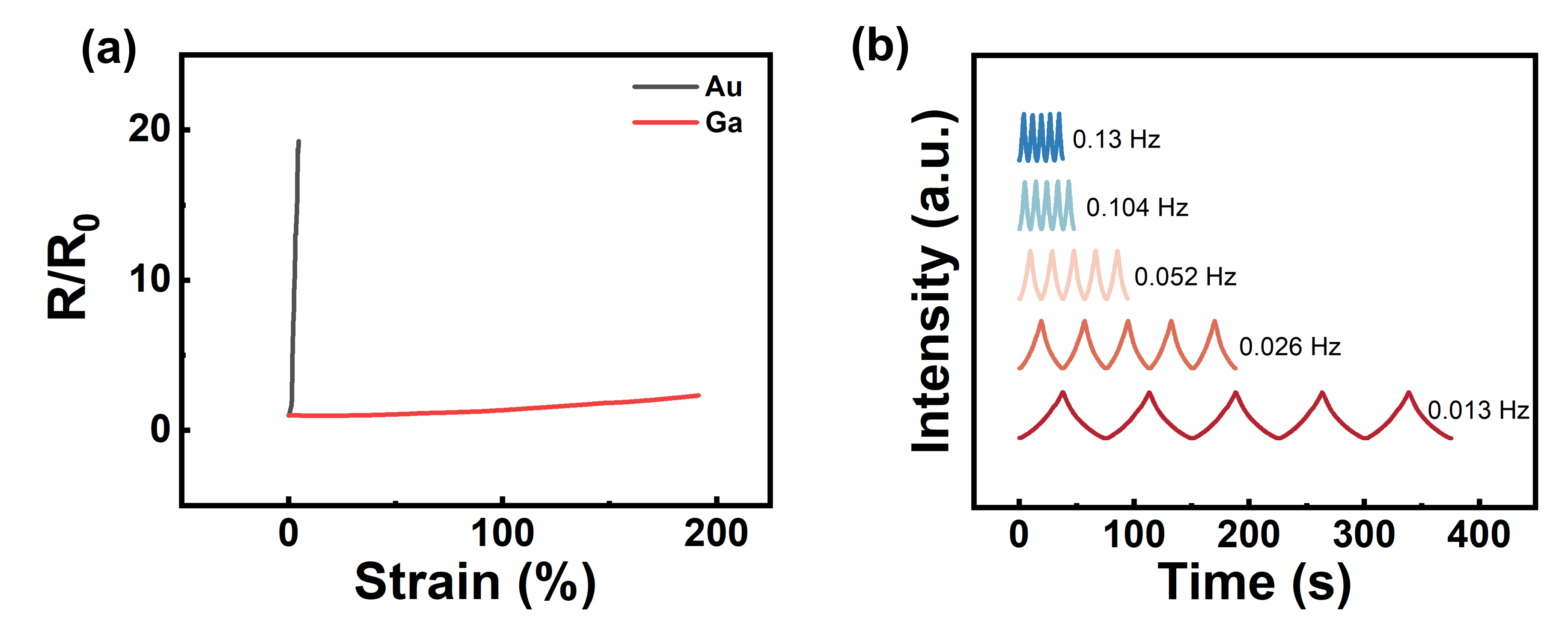


**Figure S12.** Electromechanical performance of stretchable LM based electrodes. (a) Resistance change (*ΔR/R₀*) comparison between LM and Au electrodes under uniaxial strain. (b) Frequency response under cyclic stretching.


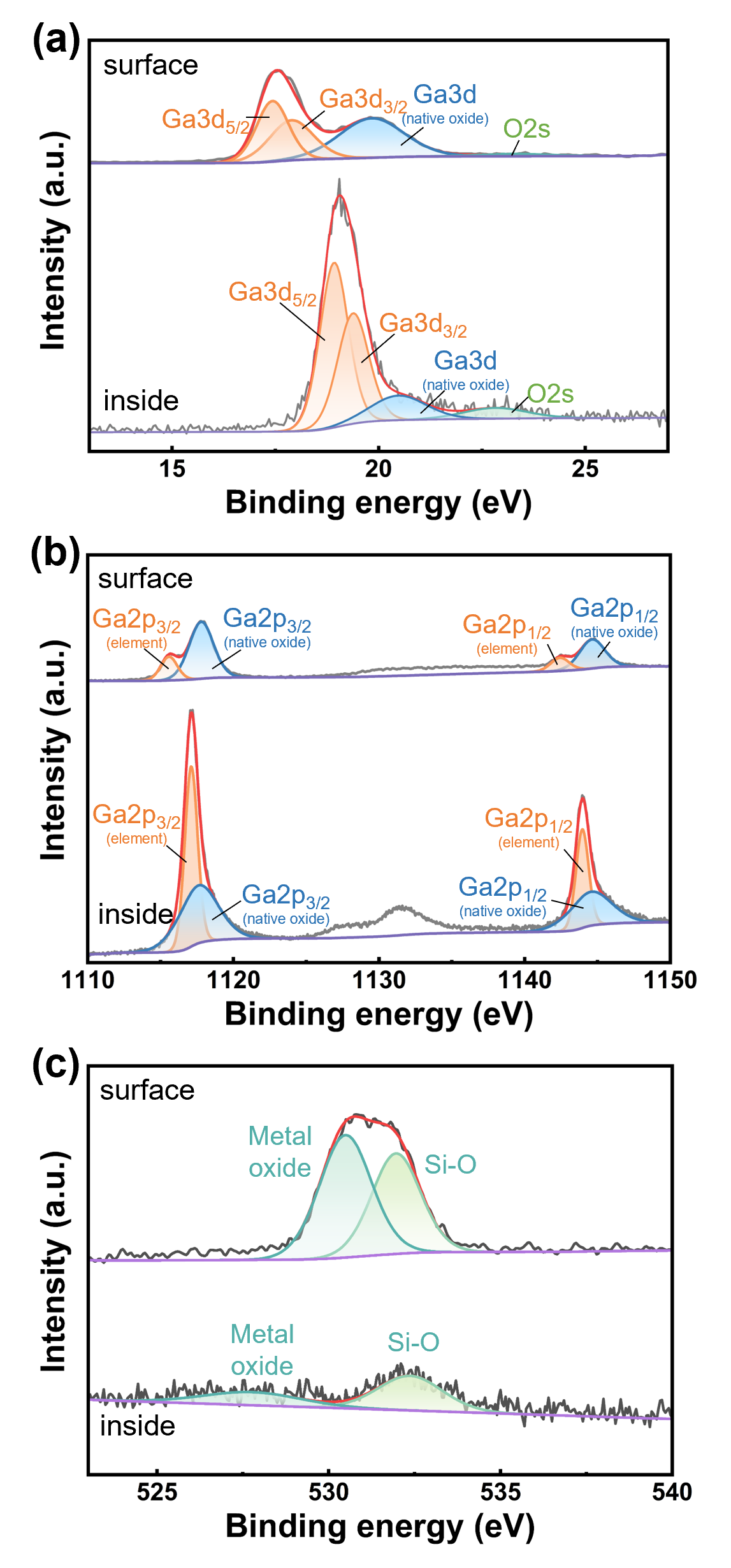
**Figure S13.** XPS analysis of the LM film surface and its interior. The interior profile was obtained after removing surface layers by Ar⁺ ion sputtering (4 keV, 4 min). (a) Ga 3d, (b) Ga 2p, and (c) O 1s core levels are presented. (The top curve corresponds to the as-prepared surface, while the bottom curve corresponds to the sputtered interior.


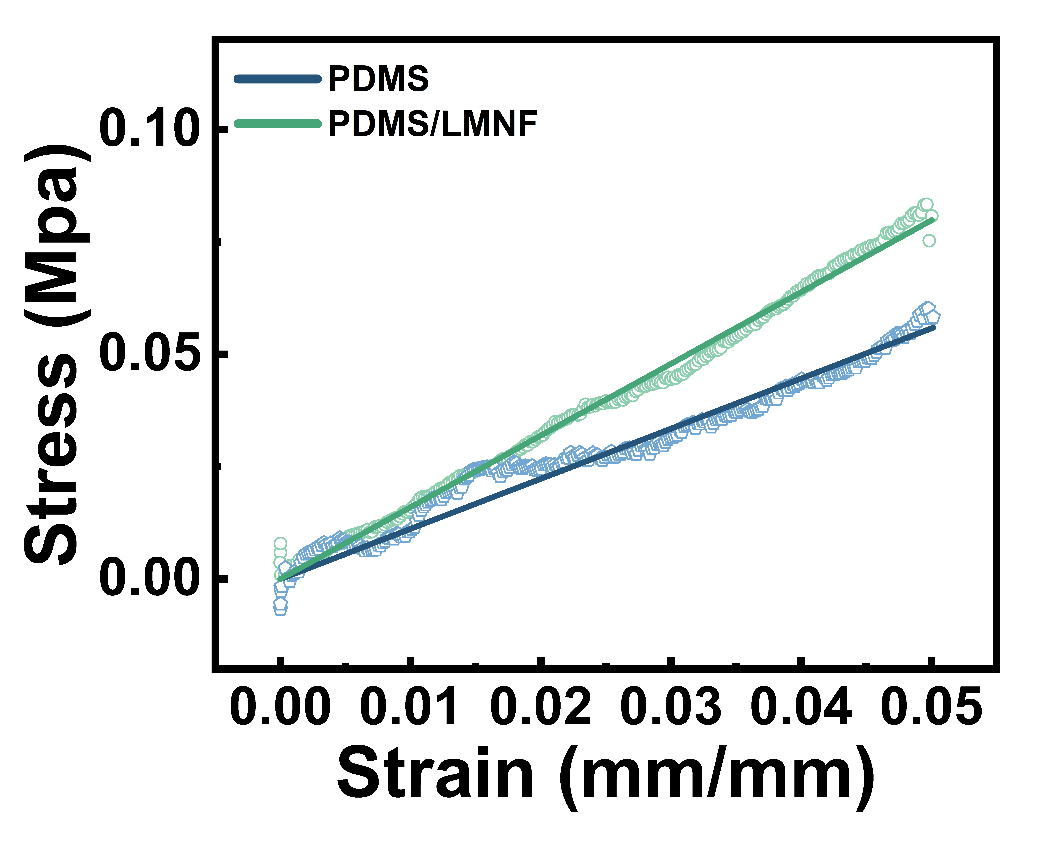


**1.596±0.01**

**1.11±0.008**

**Figure S14.** Comparison of modulus values before and after the deposition of LM films.


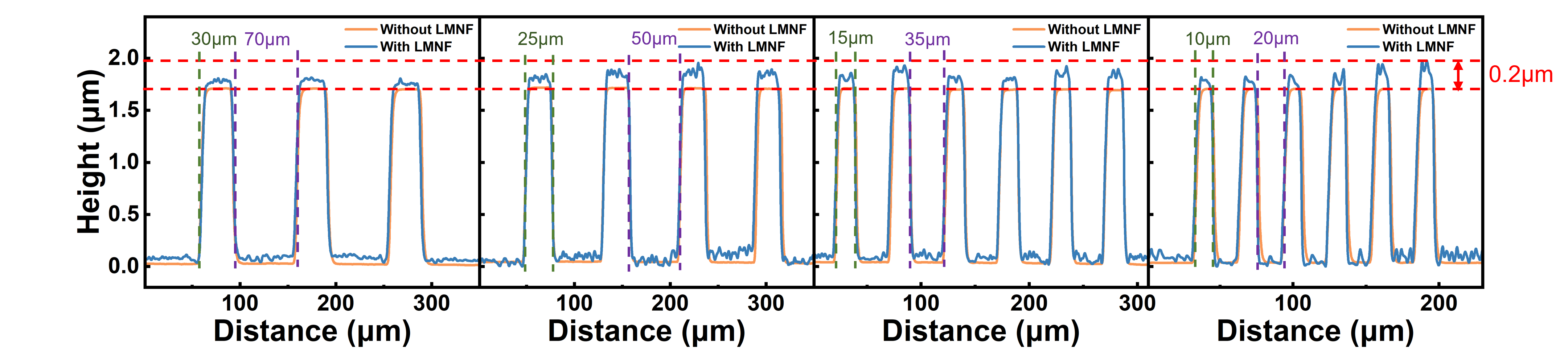


μ

μ

μ

μ

**Figure S15.** Periodic Undulating Surface Profile Characteristics Before and After Electrode Attachment.


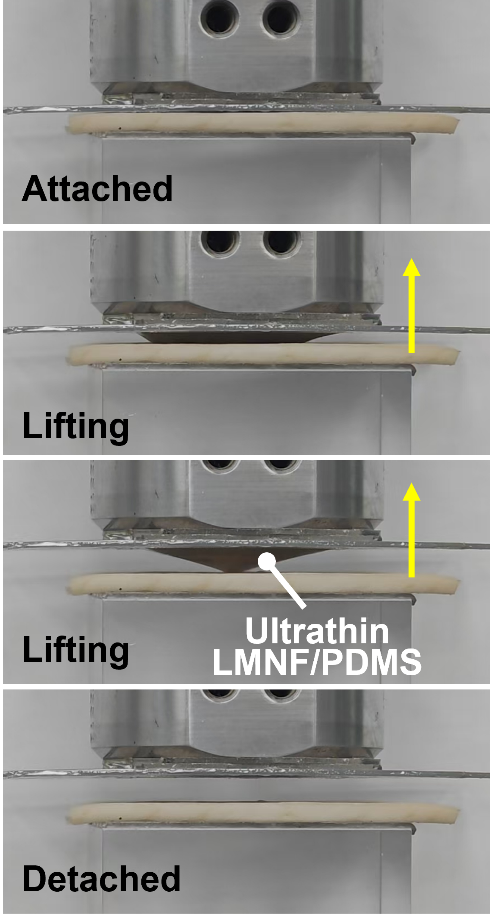


1cm

**Figure S16.** Photograph illustrating the separation process of the ultra-thin LMNF/PDMS electrode from pig skin during adhesion-separation experiments.


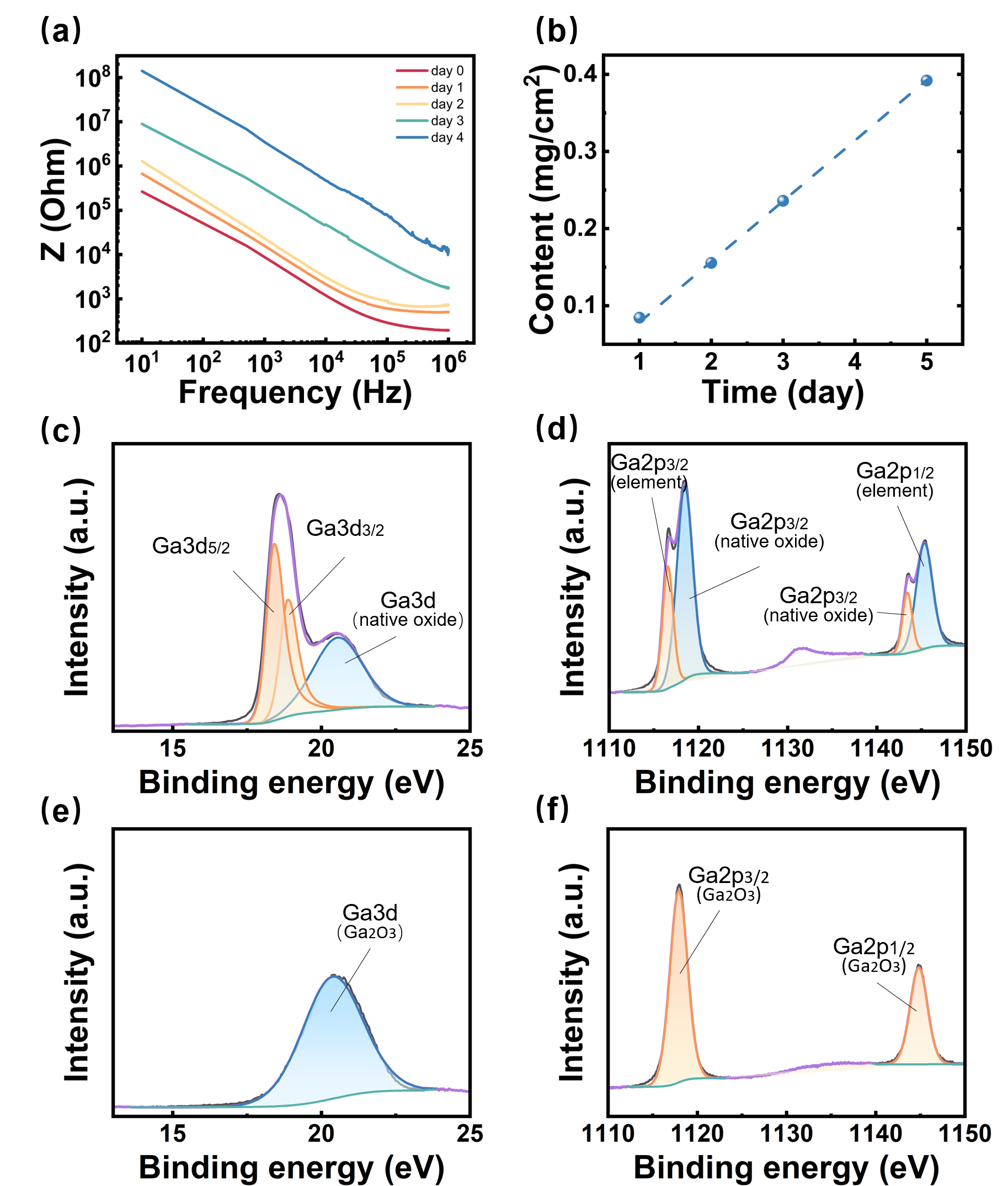


**Figure S17.** Long-term stability, ion release profile, and surface chemistry evolution of the LMNF/PDMS electrode in artificial sweat. (a) Evolution of the electrode-skin contact impedance spectra before and after immersion in 0.9% NaCl artificial sweat for 1, 2, 3, and 4 days. (b) Cumulative release amount of Ga³⁺ ions measured via ICP-OES from a 2 cm × 2 cm electrode after different immersion periods. (c, d) XPS spectra of the (c) Ga 3d and (d) Ga 2p core levels for the as-prepared film. (e, f) Corresponding (e) Ga 3d and (f) Ga 2p XPS spectra after 3 days of immersion, indicating the chemical transformation of the surface oxide layer.


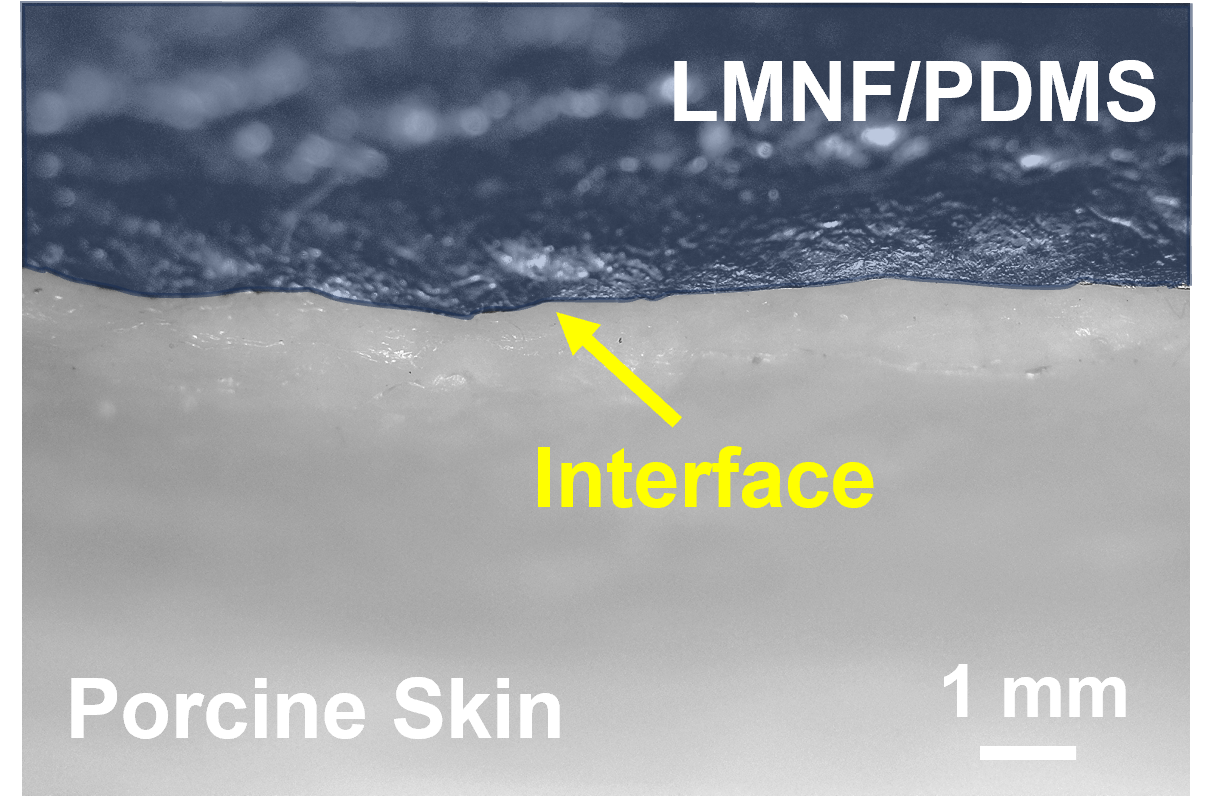


**Figure S18.** Optical photograph of the interface between the LMNF/PDMS electrode and rough porcine skin.

**
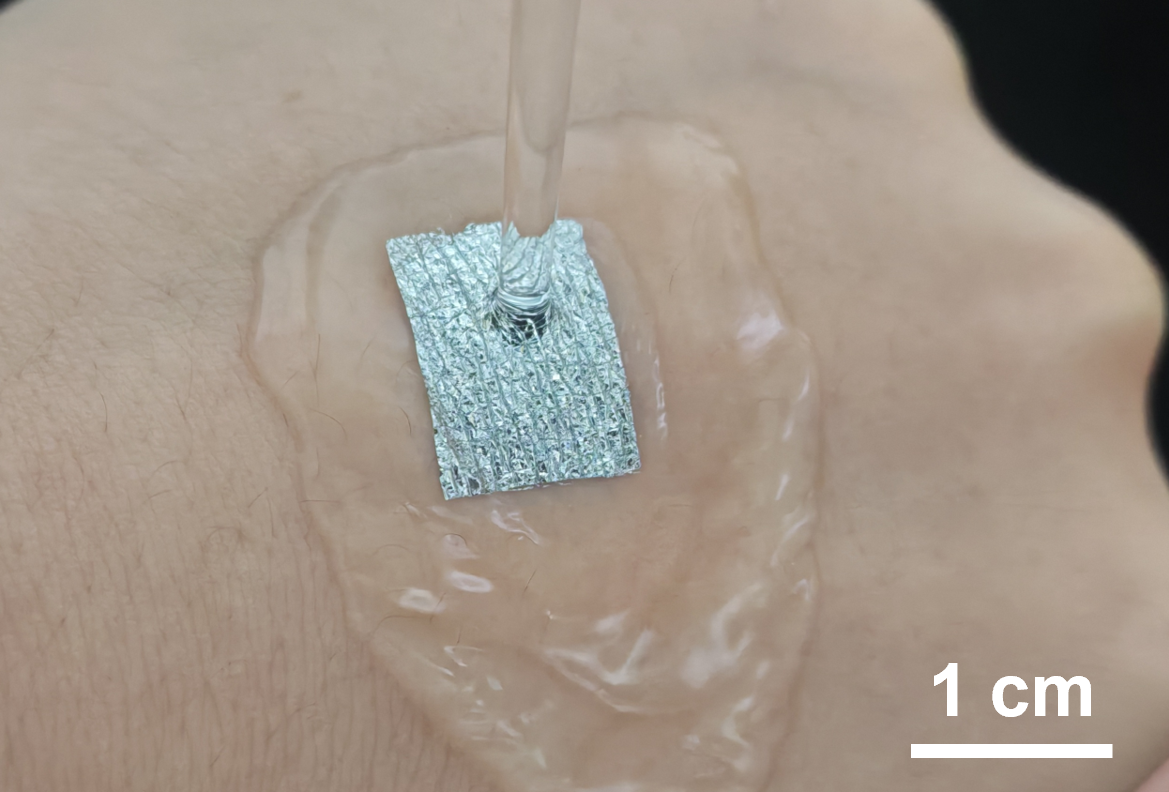
Figure S19**. Demonstration of the water-resistant capability of the ultra-thin LMNF/PDMS electrode adhered to the skin surface.


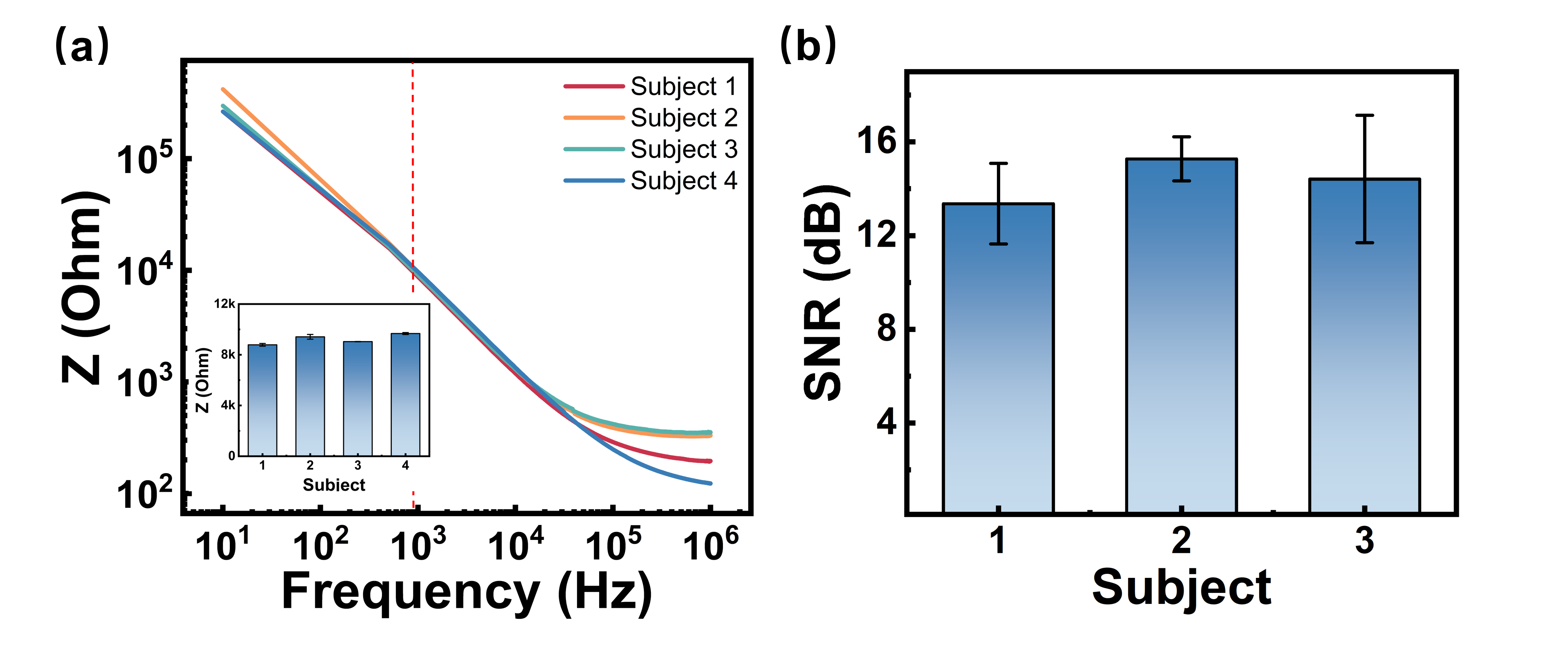


**Figure S20.** Consistency of electrode performance across multiple subjects. (a) Electrode-skin interfacial impedance spectra measured from four different subjects. The inset shows the impedance magnitude at the key frequency of 10 Hz. (b) Statistical distribution of the signal-to-noise ratio (SNR) of EMG signals acquired during hand gripping at 6 kg force from three subjects.

**
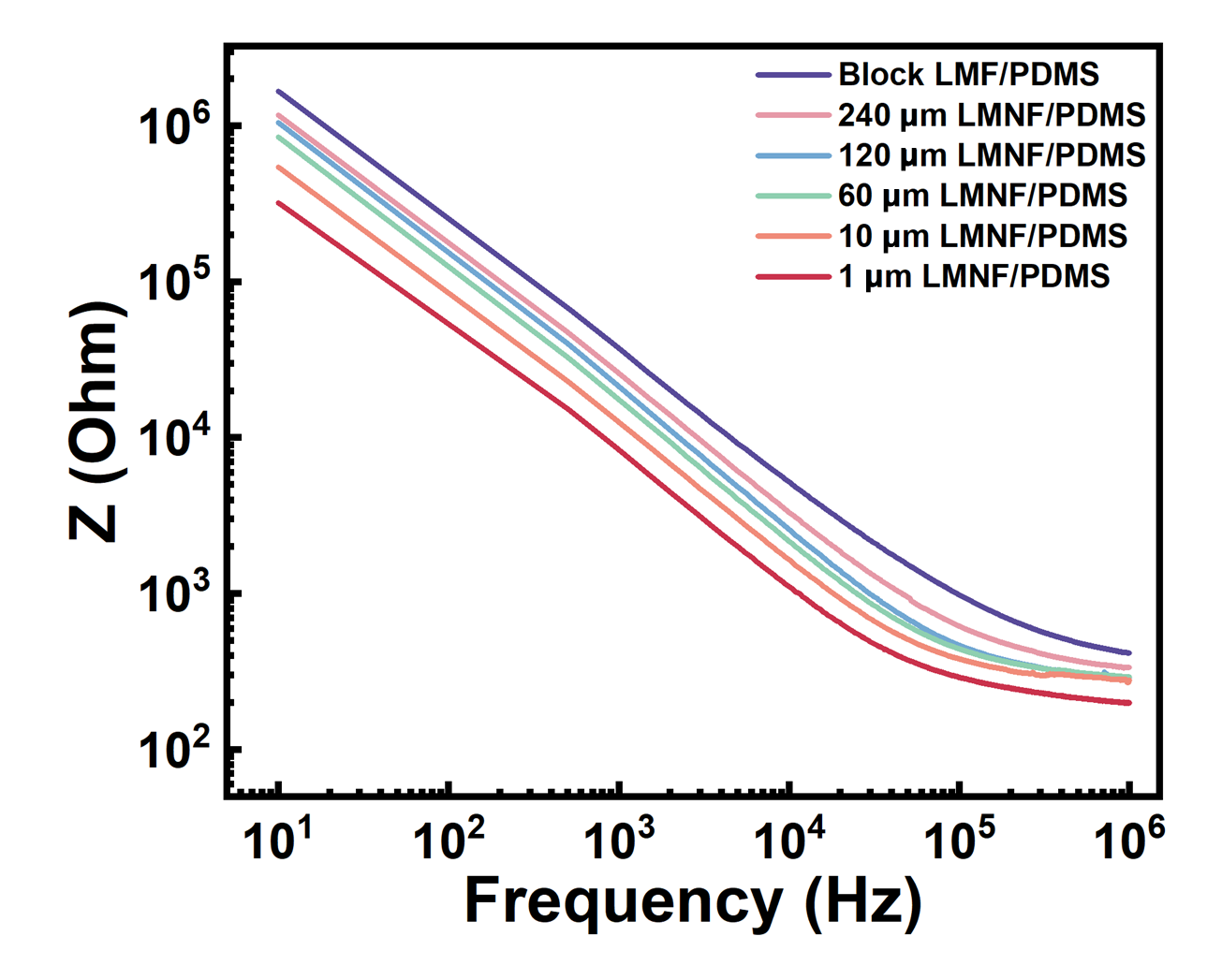
**

**Figure S21.** Skin-electrode interface impedance versus electrode thickness (Block, 240 μm, 120 μm, 60 μm, 10 μm, 1 μm).


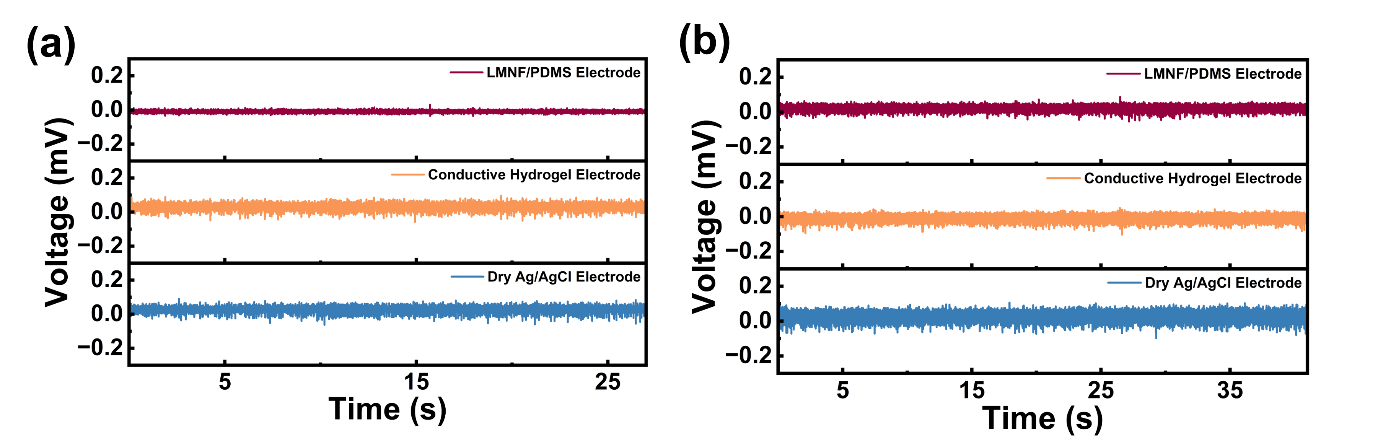


**Figure S22**. Baseline noise characterization under static (a) and dynamic (b) conditions**.**


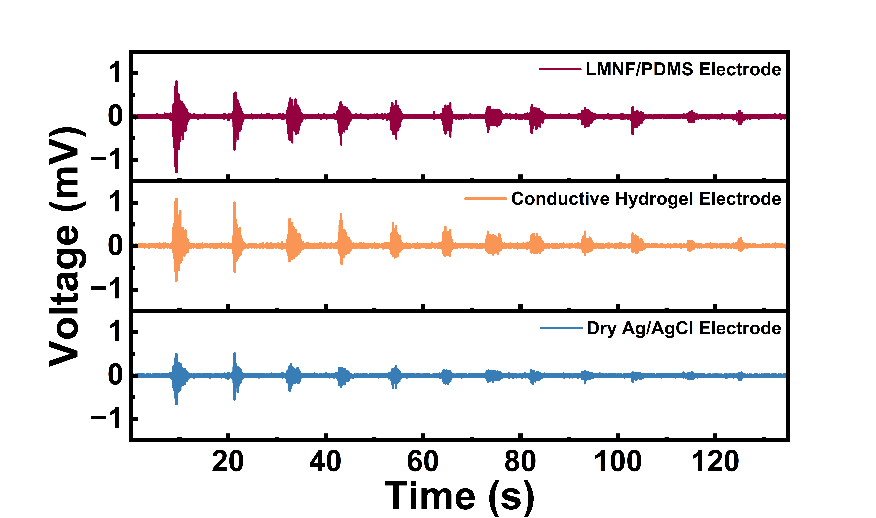


**Figure S23.** Comparison of EMG signal acquisition from the forearm muscle under 1 kg to 12 kg grip force using the ultra-thin LMNF/PDMS electrode, commercial dry electrodes, and conductive hydrogel electrodes.


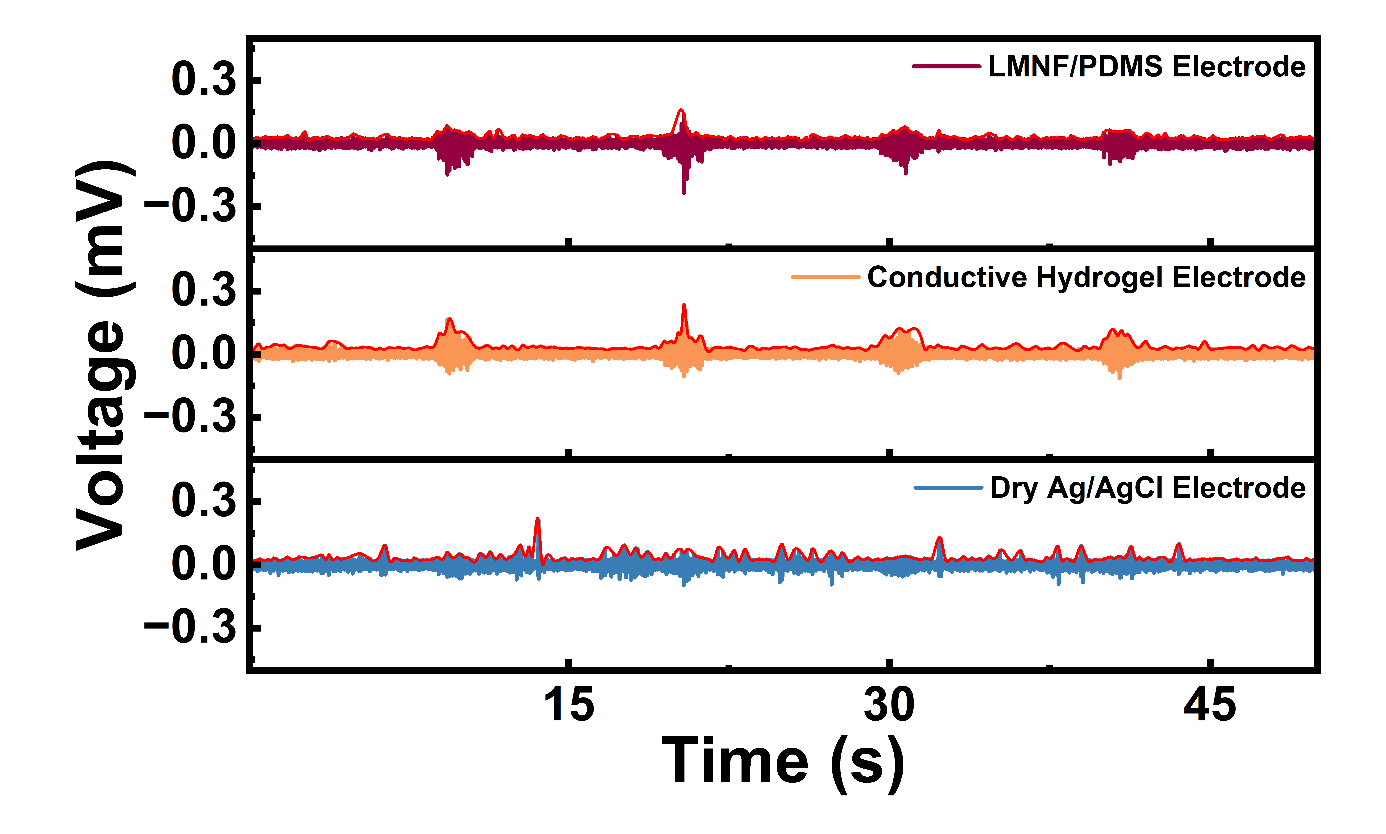


**Figure S24.** Comparison of EMG signal acquisition from the forearm muscle under 0.8 kg grip force using the ultra-thin LMNF/PDMS electrode, commercial dry electrodes, and conductive hydrogel electrodes.

**(c)**


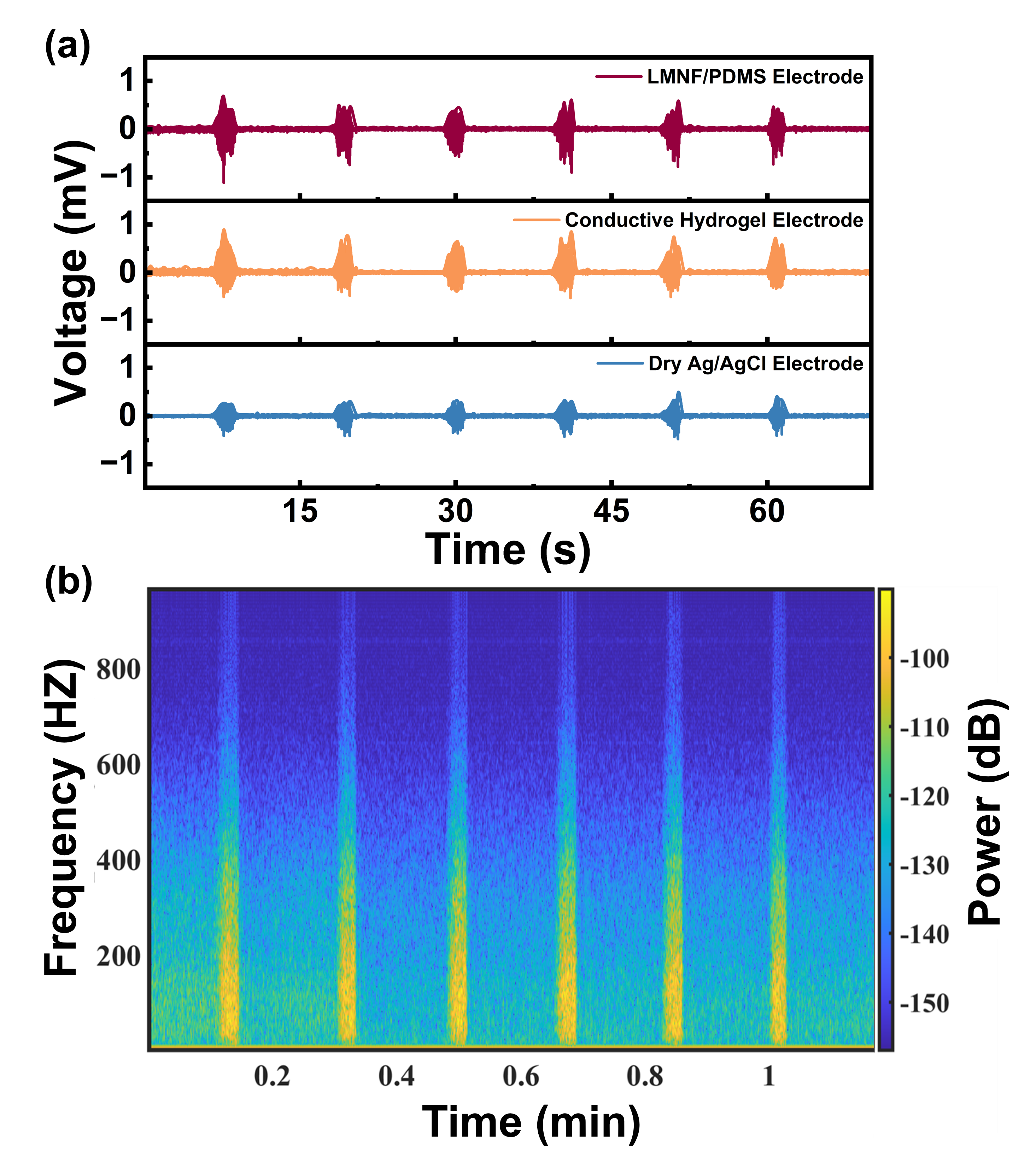

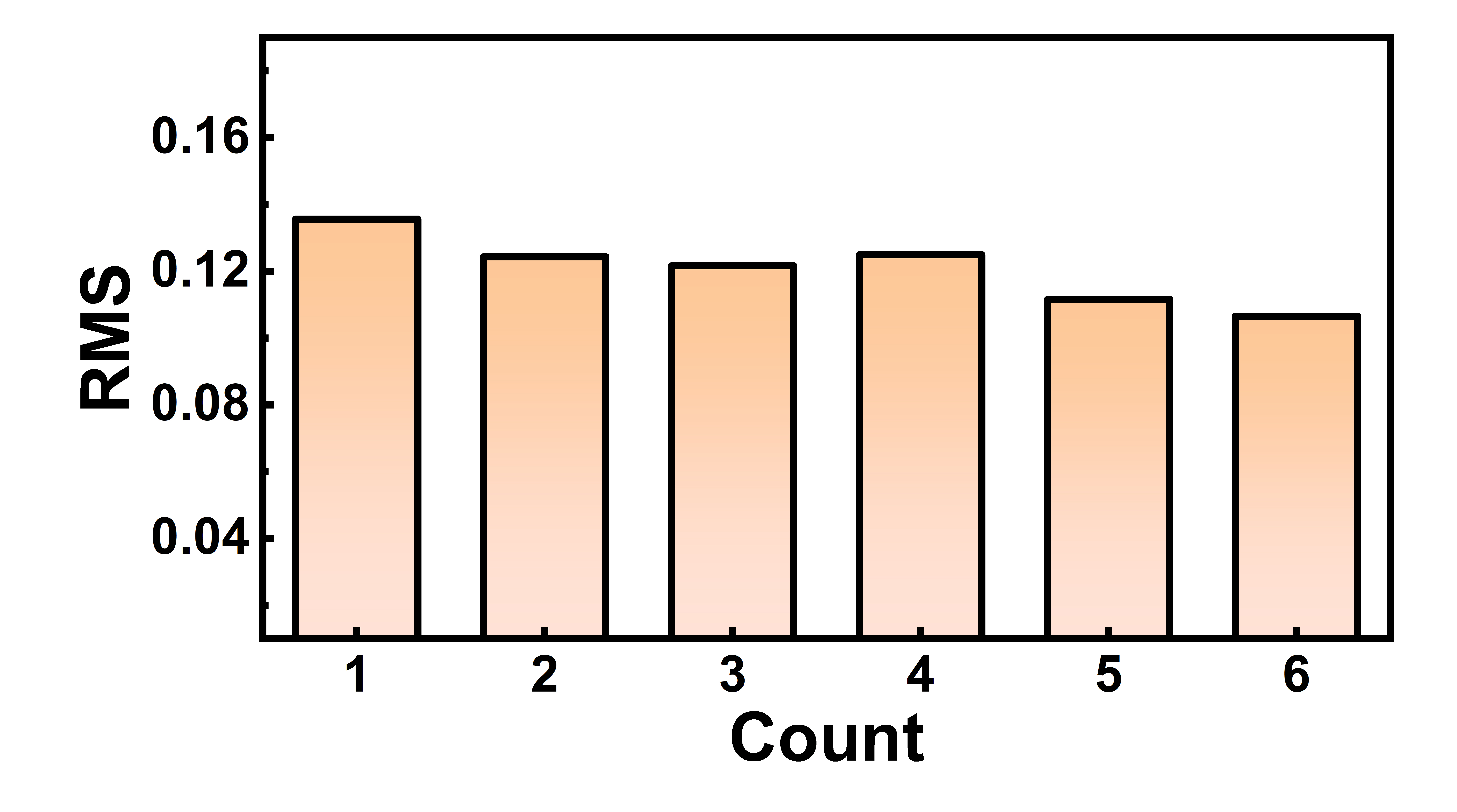


**Figure S25.** EMG signal characteristics during repetitive gripping. (a) Six consecutive grip cycles recorded by electrodes on forearms flexors. (b) Corresponding frequency spectrum (LMNF/PDMS electrodes). (c) Statistics of root mean square values of electromyographic signals under six repeated movements


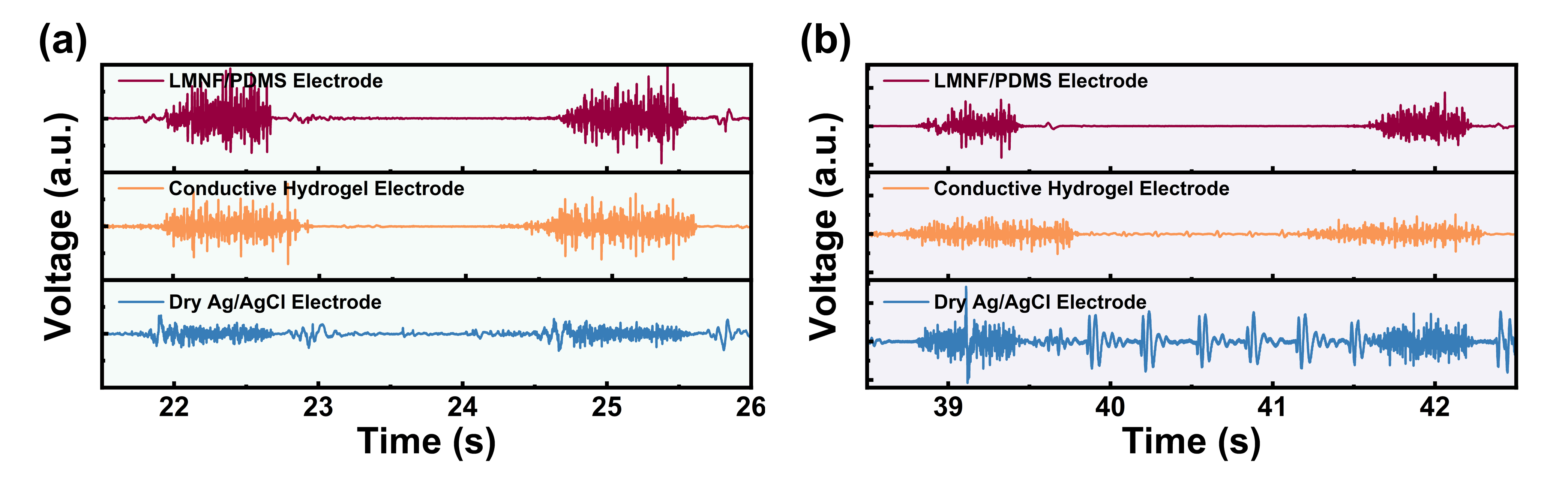


**Figure S26.** Magnified views of EMG signal details under different conditions (corresponding to Figure 5e). (a)Undisturbed signal detail. (b) 3 Hz perturbed signal detail.


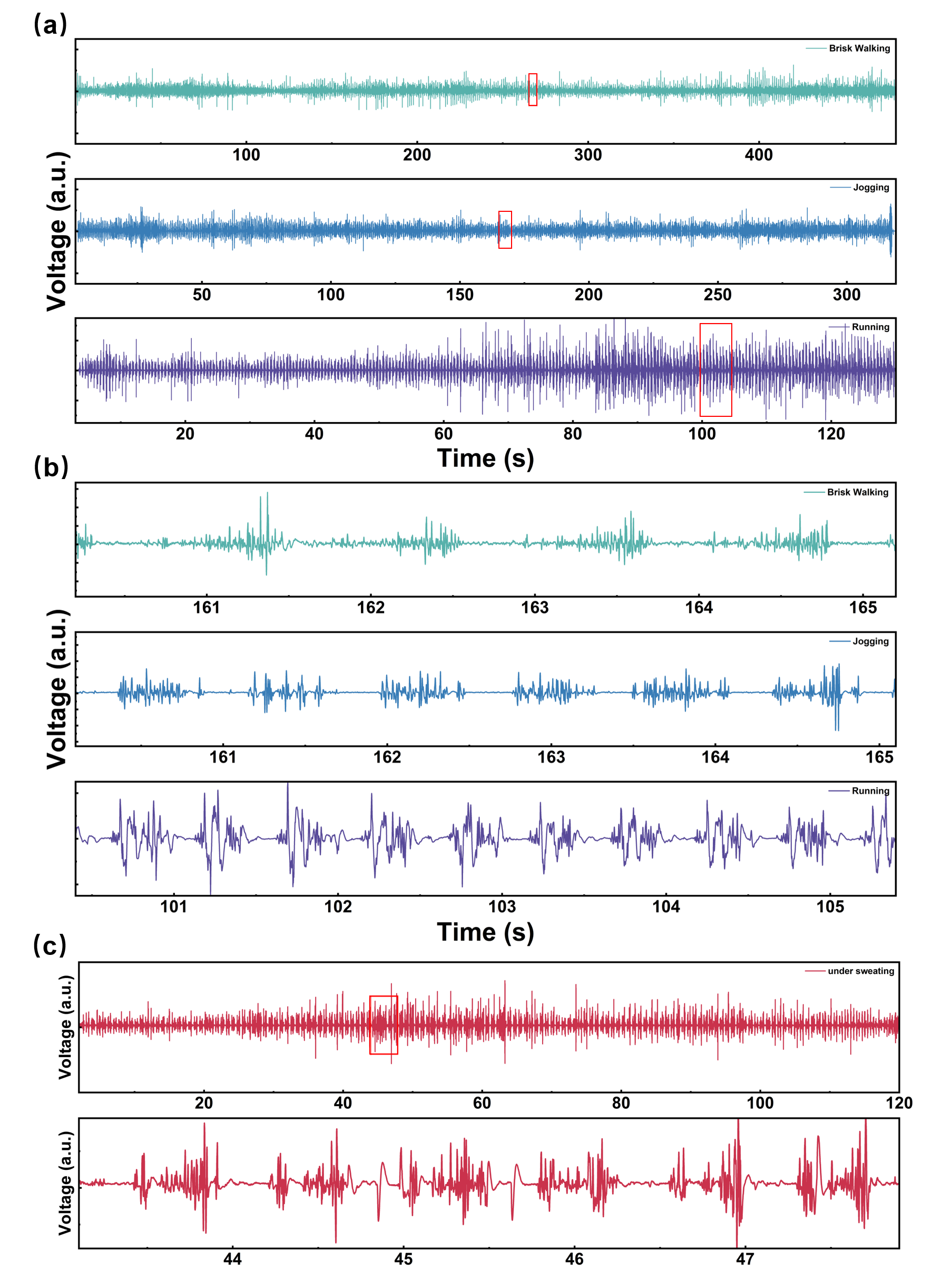


**Figure S27.** EMG signal quality during dynamic motions and under sweaty conditions. (a) Representative raw EMG signals acquired from the biceps brachii muscle during walking, jogging, and running. (b) A magnified view of the selected region in (a), highlighting the clear signal morphology under dynamic conditions. (c) EMG signal and its corresponding magnified view recorded during jogging under profuse sweating.


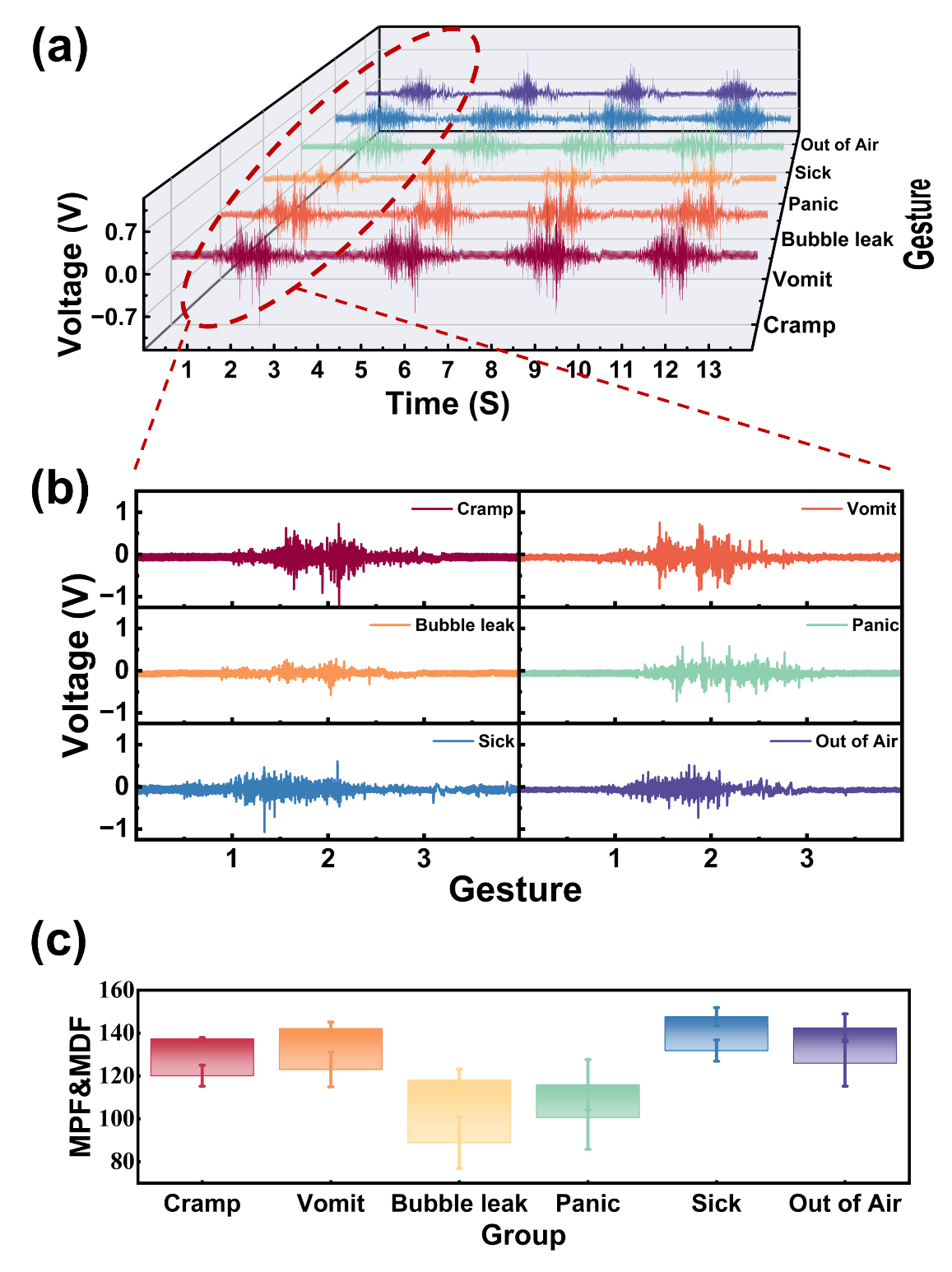


**Figure S28.** EMG signatures of six standard diving hand signals. (a) EMG signals for six repeated motions (four cycles shown). (b) Zoomed-in view of EMG signals during a single motion cycle. (c) Statistical comparison of mean power frequency (MPF, upper) and median frequency (MDF, lower ) across the six motion types.

**
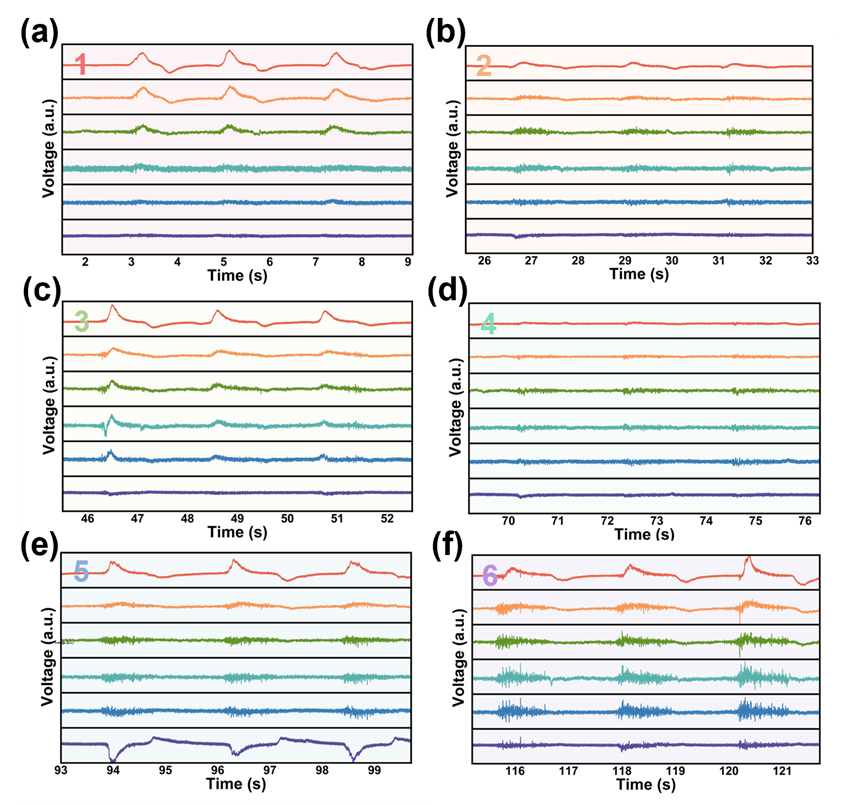
**

**Figure S29.** High-fidelity sEMG acquisition using a patterned six-channel electrode array. (a)-(f) Magnified views of the sEMG signals from the time windows indicated in Figure 5h, showing three representative repeats for each of the six gestures.


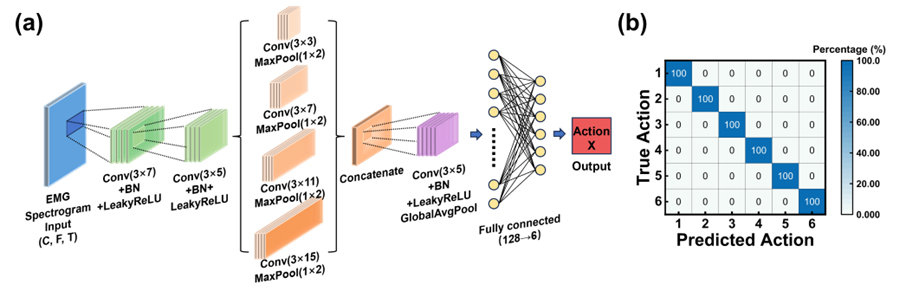


**Figure S30.** Architecture and performance of the sEMG-based gesture recognition system. (a) Schematic illustration of the multi-level convolutional neural network (CNN) framework used for gesture classification. (b) Offline recognition accuracy of the six gestures achieved by the proposed CNN model.


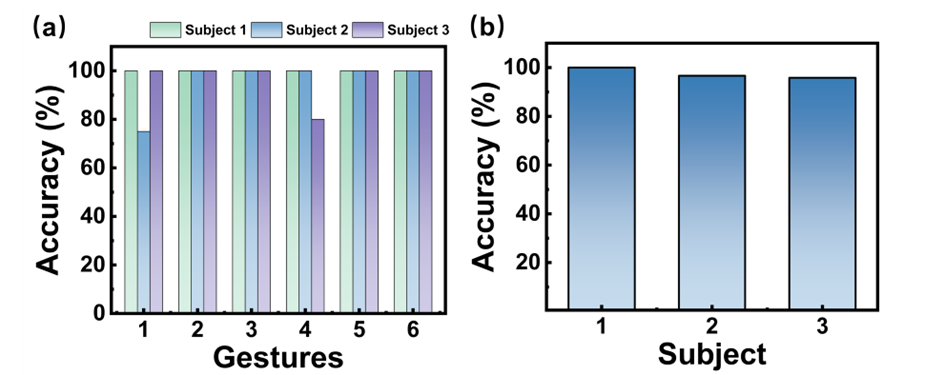


**Figure S31.** Gesture recognition performance across multiple subjects. (a) The recognition accuracy for six distinct hand gestures from three individual subjects. (b) The overall (aggregated) recognition accuracy averaged across all three subjects and all gestures.


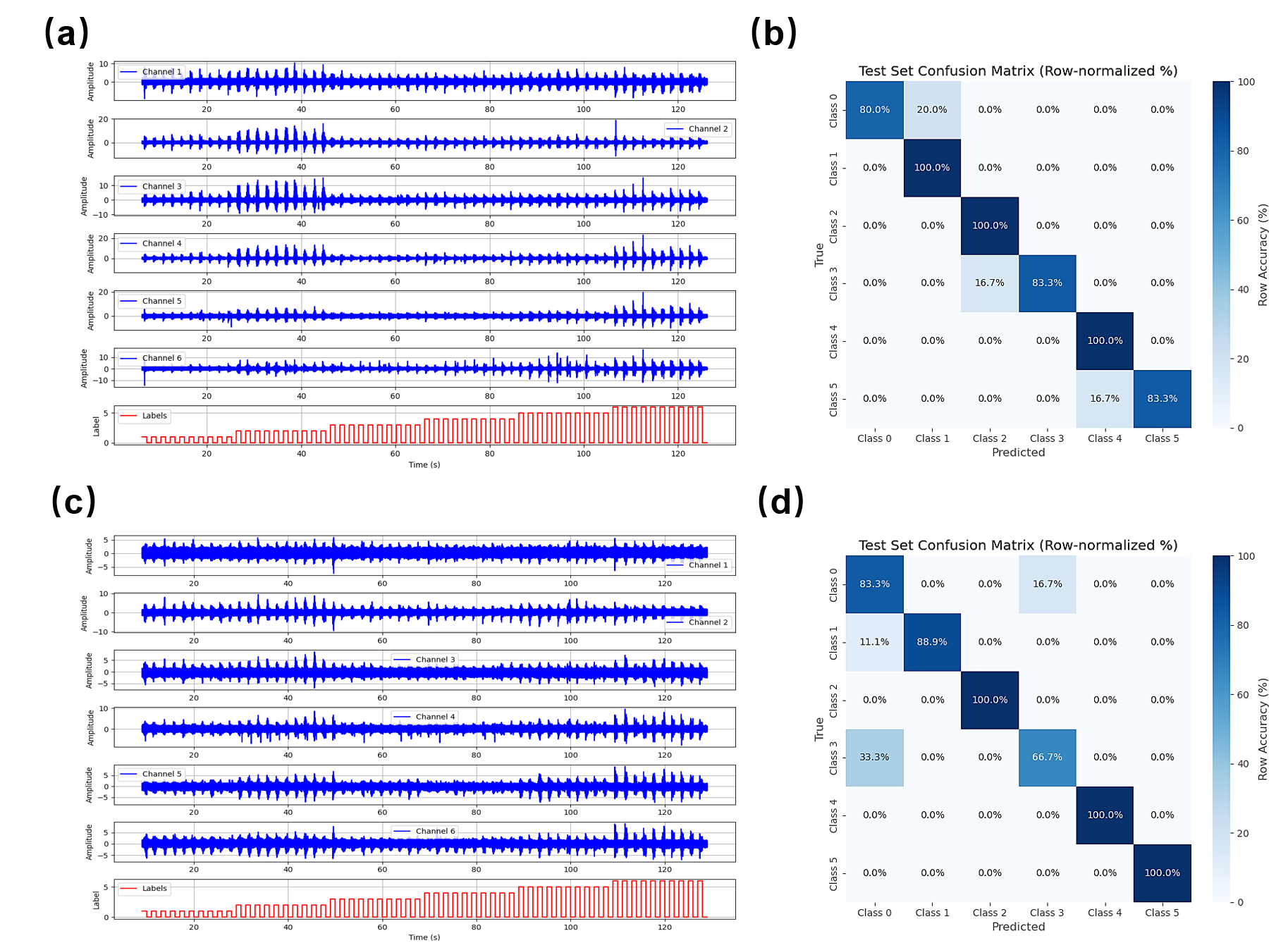


**Figure S32.** Impact of electrode placement misalignment on gesture recognition performance. (a, c) Representative raw EMG signals recorded from the first dorsal interosseous muscle when the electrode was deliberately misaligned by (a) 5 mm and (c) 10 mm along the muscle fiber direction. (b, d) The corresponding gesture recognition accuracy from a machine learning classifier under (b) 5 mm and (d) 10 mm misalignment conditions.


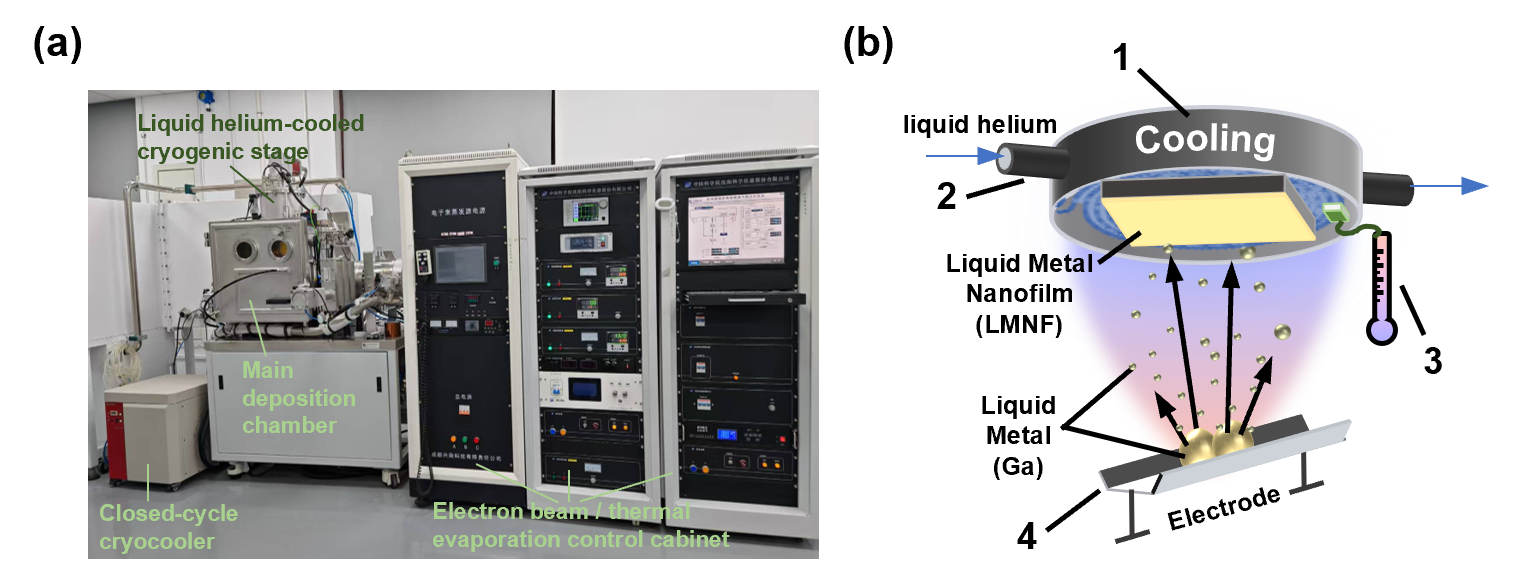


**Figure S33.** Custom multi-field physical vapor deposition (PVD) system. (a) Photograph of the complete setup, showing the integration of major components. (b) Schematic diagram illustrating the setup: (1) Substrate holder with integrated cooling/heating stage, (2) Liquid helium inlet line, (3) Electrical feedthroughs for heating and temperature sensing, (4) Thermal evaporation source (tungsten boat).

**Table S1.** Performance comparison of our LMNF/PDMS electrode with other reported ultrathin electrodes.

| Materials | Thickness  (μm) | Impedance  (KΩ) | Conductivity  (S/m) | Modulus  (Mpa) | Stretchability  (%) | Ref. |
| --- | --- | --- | --- | --- | --- | --- |
| Au/PDMS | 1.3 | - | 1.58×10^6^ | - | 300 | [11] |
| Ag/PET/PDMS/  MWCNT | 284.7 | 524  (1 Hz) | 3297.2 | - | - | [20] |
| Hydrogel/carbon nanomaterials | 115 | 167  (1000 Hz) | 35 | 0.01 | 1000 | [44] |
| LM/SBS | 2000 | - | 1.8×10^6^ | - | 1800 | [50] |
| PEDOT:PSS/PVA/d-sorbitol | 2.37 | ~11  (1000 Hz) | 3330 | 160 | 40 | [51] |
| LM/TPU | 50 | - | 3.8×10^5^ | - | 570 | [52] |
| PEDOT:PSS/  Graphene | 0.100 | 32  (100 Hz) | 4142 | 0.640 | 40 | [53] |
| PEDOT:PSS/WPU/d-sorbitol | 0.100 | 82  (10 HZ) | 545 | 50 | 30 | [54] |
| MPs+PDMS/LM  +PDMS | 200 | 47.23  (150 Hz) | 6.12×10^4^ | - | 100 | [55] |
| AgNWs/PA6 | 0.125 | - | 1.932×10^6^ | - | 50 | [56] |
| AgMPs/Ecoflex | 10 | 43  (10 Hz) | 10^4^ | 1 | - | [57] |
| AgNWs/SEBS | 100 | 50  (10 Hz) | 1.1×10^6^ | 0.2 | 150 | [58] |
| **Ga/PDMS** | **1.1** | **8.5**  **(1000Hz)** | **3.25×10^6^** | **1.596** | **190** | **This work** |

**Thermodynamic Analysis of Nucleation Kinetics**

1. Critical Nucleation Parameters

The free energy change ($\text{∆G}$) for the formation of a spherical nucleus with radius “$\text{r}$” is given by:

$\text{∆G}\text{ }\text{=}\text{ (}\text{4/3}\text{)π}\text{r}\text{³}\text{∆G}_{\text{v}}\text{+}\text{ }\text{4}\text{πr²γ}$ (1)

where:

$\text{∆}\text{G}_{\text{v}}\text{ }\text{= (k}\text{T}\text{/}\text{W}\text{) ln(}\text{P}\text{v}\text{/P}\text{s}\text{)}$ (2)

represents the volumetric free energy difference, $\gamma$is the interfacial energy.

The critical nucleus radius ($\text{r}^{\text{*}}$) and corresponding activation energy barrier ($\text{∆}\text{G}^{\text{*}}$) derived by minimizing $\text{∆G}$:

$\text{r}^{\text{*}}\text{ = -2}\text{γ/}\text{∆G}_{\text{v}}$ (3)
$\text{∆}\text{G}^{\text{*}}\text{ }\text{=}\text{ 16π}\text{γ}\text{³/(3}\text{∆G}_{\text{v}}^{\text{²}}\text{)}$ (4)

2. Temperature Dependence

Near the equilibrium temperature ($\text{T}\text{e}$), $\text{∆G}_{\text{v}}$ scales linearly with undercooling$\text{(∆T}\text{=}\text{ T }\text{-}\text{ }\text{T}_{\text{e}}\text{)}$:

${\text{∆}\text{G}\text{v}}_{\text{(T)}}\text{ ≈ }\text{∆H}_{\text{(}\text{T}_{\text{e}}\text{)}}\text{∆T/}\text{T}_{\text{e}}$ (5)

The temperature derivatives of $\text{r}^{\text{*}}$ and $\text{∆}\text{G}^{\text{*}}$ reveal key trends:

${\text{(}\text{∂}\text{r}^{\text{*}}\text{/∂T}\text{)}}_{\text{R}}\text{ }\text{= }\text{r}^{\text{*}}\text{(}\frac{1}{\text{γ}}\frac{\text{∂γ}}{\text{∂T}} \text{-}\text{ }\frac{1}{\text{∆G}_{\text{v}}}\frac{\text{∂}\text{∆G}_{\text{v}}}{\text{∂T}}\text{)}\text{ }\text{> 0}$ (6)
$\text{(}\text{∂∆}\text{G}^{\text{*}}\text{/∂}\text{T}\text{)}\text{R}\text{ }\text{=}\text{ ∂}\text{∆G}_{\text{v}}\text{ (}\frac{3}{\text{γ}}\frac{\text{∂γ}}{\text{∂T}}\text{ -}\text{ }\frac{2}{\text{∆G}_{\text{v}}}\frac{\text{∂}\text{∆G}_{\text{v}}}{\text{∂T}}\text{) }\text{＞}\text{ 0}$ (7)

3. Implications for Microstructure Formation

As the temperature decreases:

1. Reduced critical radius (from Eq. 6): Lower $\text{r}^{\text{*}}$ enables nucleation at smaller length scales.
2. Lower energy barrier (from Eq. 7): $\text{∆}\text{G}^{\text{*}}$ decreases, increasing nucleation rate (𝐼 ∝ exp($\text{-∆}\text{G}^{\text{*}}\text{/kT}$)).
3. Refined grain morphology: Higher nucleus density promotes a fine-grained, continuous film structure.
